# Characterization of novel LncRNA *P14AS* as a protector of *ANRIL* through AUF1 binding in human cells

**DOI:** 10.1186/s12943-020-01150-4

**Published:** 2020-02-27

**Authors:** Wanru Ma, Juanli Qiao, Jing Zhou, Liankun Gu, Dajun Deng

**Affiliations:** Key Laboratory of Carcinogenesis and Translational Research (Ministry of Education/Beijing), Division of Etiology, Peking University Cancer Hospital & Institute, Fu-Cheng-Lu #52, Haidian District, Beijing, 100142 China

**Keywords:** lncRNA, *CDKN2A*, *P14AS*, *ANRIL*, *P16*, AUF1, Colon cancer

## Abstract

**Background:**

The *CDKN2A/B* locus contains crucial tumor suppressors and a lncRNA gene *ANRIL*. However, the mechanisms that coordinately regulate their expression levels are not clear.

**Methods:**

Novel RNAs transcribed from the *CDKN2A* gene were screened by *CDKN2A*-specific RNA capture deep-sequencing and confirmed by Northern blotting and clone-sequencing. Long non-coding RNA (lncRNA) binding proteins were characterized by RNA pull-down combined with mass spectrometry and RNA immunoprecipitation. LncRNA functions in human cells were studied using a set of biological assays in vitro and in vivo.

**Results:**

We characterized a novel lncRNA, *P14AS* with its promoter in the antisense strand of the fragment near *CDKN2A* exon 1b in human cells. The mature *P14AS* is a three-exon linear cytoplasmic lncRNA (1043-nt), including an AU-rich element (ARE) in exon 1. *P14AS* decreases AUF1-*ANRIL/P16* RNA interaction and then increases *ANRIL/P16* expression by competitively binding to AUF1 P37 and P40 isoforms. Interestingly, *P14AS* significantly promoted the proliferation of cancer cells and tumor formation in NOD-SCID mice in a *P16*-independent pattern. Moreover, in human colon cancer tissues, the expression levels of *P14AS* and *ANRIL* lncRNAs were significantly upregulated compared with the paired normal tissues.

**Conclusion:**

A novel lncRNA, *P14AS*, transcribed from the antisense strand of the *CDKN2A/P14* gene, promotes colon cancer development *by cis* upregulating the expression of oncogenic *ANRIL*.

## Background

Three important tumor suppressor genes (*P16*^*INK4A*^, *P14*^*ARF*^ and *P15*^*INK4B*^) and the oncogenic lncRNA *ANRIL* (*CDKN2B-AS1*) resides in the human *CDKN2A/B* locus at chromosome 9p21 [1]. P16 and P15 proteins target CDK4/6 through the CDK4/6-RB-E2F pathway, and inactivation of *P15* and/or *P16* allows cells to escape cell cycle arrest in G1 while P14 protein binds to MDM2 and results in P53 activation. While *ANRIL* was reported to downregulate *P15* and *P16* expression by interacting with components of polycomb repressive complex-1/-2 [2–5], *ANRIL* was also found to be coordinately transcribed with *P16* in cancer cells and transcriptionally repressed by *P16* DNA methylation [6]. This gene locus is frequently inactivated in cancer genome by somatic copy-number deletion and DNA methylation, leading to familial pancreatic cancer and melanoma.

AUF1 is an essential RNA binding protein that promotes the decay of many cancer-related RNAs, including *P16*, *c-MYC*, *NEAT1* [7–10]. In this study, we designed a probe set to capture all possible transcripts from the *CDKN2A* locus and performed an extra-deep sequencing (CDKN2A RNACap-Seq) to identify given and novel RNAs from this gene and flanking regions. We characterized, for the first time, a novel lncRNA called *P14AS* (NCBI GenBank MK574077) transcribed from the antisense strand of the fragment around exon 1β of the *CDKN2A* gene. We found that AUF1 binds to *P14AS* and the AUF1 binding of the ARE (AU-rich element) in exon 1 of the *P14AS* gene increases *ANRIL/P16* level. Furthermore, a significantly higher level of *P14AS* exists in colon cancers compared to paired normal tissues and that *P14AS* could markedly promote the proliferation of cancer cells and tumor formation in a *P16*-independent pattern in vitro and in vivo.

## Methods

### Cell culture and tissue samples

The human cell line HEK293T was kindly provided by Professor Yasuhito Yuasa at Tokyo Medical and Dental University. HCT116 and SW480 cells were kindly provided by Professor Yuanjia Chen at Peking Union Medical College Hospital. The MCF7 cell line was kindly provided by Professor Yuntao Xie; BGC823, MGC803 and SGC7901 cells were kindly provided by Professor Yang Ke; A549 and HEK293FT cells were kindly provided by Professor Zhiqian Zhang; the HepG2 cell line was kindly provided by Professor Qingyun Zhang; the AGS cell line was kindly provided by Professor Chengchao Shou at Peking University Cancer Hospital and Institute.

The colon cancer (CC) tissues, paired normal tissues from the surgical margin (SM, > 5 cm from cancer lesion) from CC patients (*N* = 172, including 75 cases in the pilot study and 97 additional cases in the clinical association analysis), and normal colon mucosa biopsy samples from noncancer patients (*N =* 50) were collected and stored at − 70 °C at Peking University Cancer Hospital from 2004 to 2011. Research protocols were approved by the Institutional Review Board of the Peking University Cancer Hospital and Institute, China. Clinical and histopathological data for each patient were obtained according to approved institutional guidelines.

### CDKN2A-RNACap-Seq

**Fig. 1:**
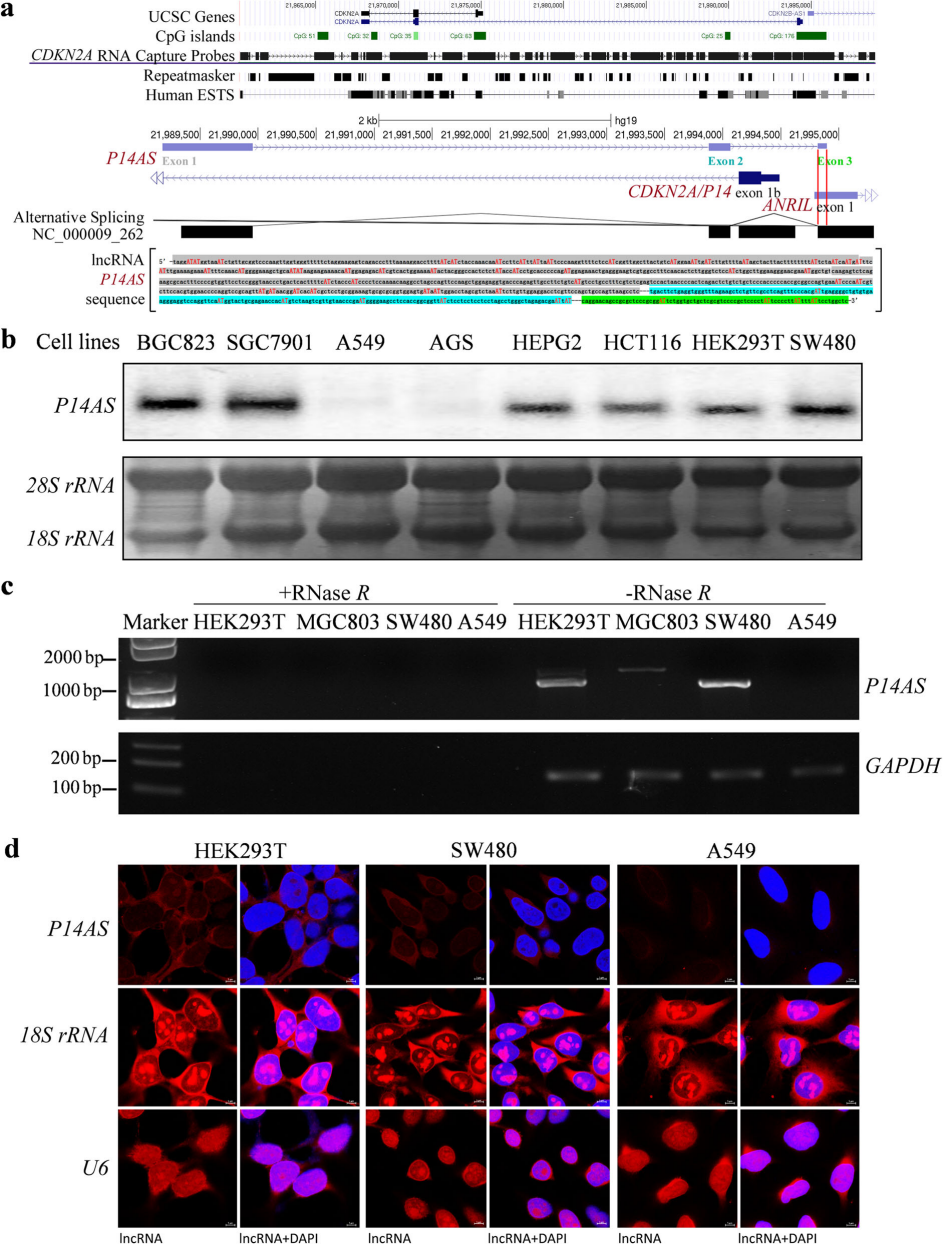
Characterization of lncRNA *P14AS* in cancer cells. **a** Illustration of the location of *CDKN2A* RNA capture probes (30 k) and lncRNA *P14AS* transcribed from the antisense strand of the sequence around exon 1β of the *CDKN2A* gene (chr9: 21,989,178-21,994,898 in the human genome GRCh37/hg19). Sequences for the three exons are highlighted with different colors: exon 1 in gray, exon 2 in blue, and exon 3 in green. AT (AU in RNA) sites are marked with red letters. **b** The expression status of *P14AS* in various human cell lines by Northern blotting. An 80-nt *P14AS*-specific probe was labeled with ^32^P for Northern blot analysis. The *18S rRNA* and *28S rRNA* were used as RNA controls. **c** cDNA synthesized from total RNA with or without RNase *R* digestion was used as the PCR template. **d** Cytoplasm *P14AS* (red) was visualized by RNA-FISH in HEK293T and SW480 cells, but not in A459 cells without the *CDKN2A/B* locus. *P14AS*-specific probes were labeled with Cy3 dye. The *18S rRNA* and *U6 RNA* were used as the cytoplasm and nucleus RNA controls, respectively. Scale bar, 5 μm

Total RNA was used to synthesize complementary DNA (cDNA). In total, 3 μg of cDNA was fragmented by nebulization. The fragmented DNA was repaired, and an ‘A’ was ligated to the 3′ end. Illumina adapters were then ligated to the fragments, and the sample was size-selected with a 350–400-bp product. The size-selected product was amplified by PCR. Each sample was tagged with a unique index during this procedure, and the final product was validated using a Bioanalyzer (Agilent, USA). The amplified DNA (1 μg) was mixed with a *CDKN2A*-specific biotinylated probe panel to tile along chr9: 21,959,171-21,999,170 (30-kb; hg19; excluding repeat sequences; MyGenostics GenCap Enrichment Technologies, Beijing, China; Fig. 1a), held at 65 °C with the PCR lid heat on for 22 h for hybridization. The hybridized DNA was heated at 95 °C for 7 min and 65 °C for 2 min in the PCR machine. Then, 23 μL of the 65 °C prewarmed Buffer HY (MyGenostics, Beijing, China) was added to the mix. 50 μL of MyOne beads (Life Technology, USA) was washed in 500 μL 1× binding buffer 3 times and resuspended in 80 μL 1× binding buffer. Then, 64 μL 2× binding buffer was added to the hybrid mix and transferred to the tube with 80 μL MyOne beads. The mixture was rotated for 1 h on a rotator. The beads were then washed once with WB1 buffer at room temperature for 15 min and three times WB3 buffer at 65 °C for 15 min. The bound DNA was then eluted with Buffer Elute. The eluted DNA was finally amplified for 15 cycles using the following program: 98 °C for 30 s (1 cycle); 98 °C for 25 s, 65 °C for 30 s, 72 °C for 30 s (15 cycles); and 72 °C for 5 min (1 cycle). The PCR product was purified using SPRI beads (Beckman Coulter) according to the manufacturer’s protocol. The enrichment libraries were sequenced on an Illumina HiSeq 2000 sequencer for paired reads of 100-bp.

Illumina clean reads were aligned to each human reference genome using the BWA program, and quality scores were recalibrated and realigned to references using the GATK software package. Duplicated reads were removed using Sequence Alignment/Map tools (SAMtools), and only uniquely mapping reads were used for variation detection. The RNA-seq datasets were uploaded into the NCBI website (GES128205).

### Northern blotting

Total RNA was electrophoresed in a formaldehyde denaturing agarose gel after extracting from cells with the Ultrapure RNA kit (Beijing Com Win Biotech Co., Ltd., China). The RNA (10 μg) was transferred to a positively charged NC film with 20× SSC buffer (3.0 M NaCl and 0.3 M sodium citrate, pH 7.0). The membrane was UV-cross-linked and incubated with ^32^P-labeled RNA probes (Table 1) generated by the Rediprime II Random Prime Labelling System according to the manufacturer’s instruction. The membrane was washed 2–3 times using 2× SSC/0.2% SDS under the condition of 65 °C for 15 min. The membrane was put into the phosphorus screen and pressed for several hours to overnight according to the signal strength.

**Table 1:** Sequences of oligo probes and primers

Assay	Oligo name	Oligo sequence (5′-3′)		Temp.
Northern blotting	*P14AS* probe	acgctgcggatcccagaggcttaactggcagctggaacgaggtcctccaacaagaatttagacgctaggtccaattatca		
RNA pull-down	*P14AS* probe #1	accaacttgggaccgcaacagatttaccata	(1–200 nt)	
	*P14AS* probe #2	gtccttttaaagggtctgactcttcctagaa	(1–200 nt)	
	*P14AS* probe #3	ggtgcaggatggtatagagagtggcccgtag	(201–400 nt)	
	*P14AS* probe #4	ctgggtcacctctccagcttggaactggcta	(401–600 nt)	
	*P14AS* probe #5	gcaggagcgatgtgatccgttatcataactg	(601–777 nt)	
	*P14AS* probe #6	aacgaggtcctccaacaagaatttagacgct	(601–777 nt)	
	Control #1	ttaacgcctcgaatcagcaa		
	Control #2	gatcttccagataactgccg		
RNA EMSA	probe#1	tttcatttgaaaagaaaattttcaaacatggggaaagctgcaatataagaagaaaacaatggagagacatcgtcactgga		
	probe#2	aatactacgggccactctctataccatcctgcacccccagatggagaaactgagggaagtcgtggcctttcaacactctt		
*P14AS* CRISPR/Cas9	gRNA #1	catgacagtaagccaaccgatgg	(HEK293T)	
	gRNA #2	gttagtggactcgagacgaaagg	(HEK293T)	
	gRNA #3	tgttgcggtcccaagttggtggg	(HCT116)	
	gRNA #4	gttagtggactcgagacgaaagg	(HCT116)	
*P14AS* promoter	gRNA#5	acaattagatgttcaactggggg	(HEK293T)	
CRISPR/Cas9	gRNA#6	cgtatcttatatagcttatgtgg		
qRT-PCR primers	*P14AS-*F1	aacggatcacatcgctcctg	254 bp	58 °C
	*P14AS-*R1	tccccattcgggttacaacg		
	*ALU-*F	gaggctgaggcaggagaatcg	87 bp	60 °C
	*ALU*-R	gtcgcccaggctggagtg		
	*GAPDH*-F	gagatggtgatgggatttc	224 bp	62 °C
	*GAPDH*-R	gaaggtgaaggtcggagt		
	*P16*-F	gctgcccaacgcaccgaata	180 bp	60 °C
	*P16*-R	accaccagcgtgtccaggaa		
	*P15*-F	agtcaaccgtttcgggaggcg	168 bp	62 °C
	*P15*-R	accaccagcgtgtccaggaag		
(q)RT-PCR primers	*ANRIL*-F	cagcagaaggtgggcagcagat	145 bp	64 °C
	*ANRIL*-R	ttcctcgacagggcaggcaggt		
	*18S rRNA*-F	gcttaatttgactcaacacggga	69 bp	58 °C
	*18S rRNA*-R	agctatcaatctgtcaatcctgtc		
RT-PCR primers	*P14AS*-F2	taggatatggtaaatctgttgcggt	1043 bp	58 °C
	*P14AS*-R2	gagccaggaataaaataaggggaat		
*P14AS* promoter primers	*P14AS-*F3	ccatgtgatttaggaagaaagtttc	1281 bp/628 bp	58 °C
	*P14AS-*R3	ttaacaacagcattattacctgggc		
PCR Out-primers	*P14AS*-F4	taggatatggtaaatctgttgcggt	1234 bp	58 °C
	*P14AS*-R4	tagggagggaggaaagacaaggaa		
PCR In-primers	*P14AS*-F5	caaacatggggaaagctgcaa	164 bp	58 °C
	*P14AS*-R5	cccttccaagccagatggag		
siRNA	*P14AS*-S1F	ggaagagucagacccuuuatt		
	*P14AS*-S1R	uaaagggucugacucuucctt		
	*P14AS*-S2F	gcuuacugucauggaaauutt		
	*P14AS*-S2R	aauuuccaugacaguaagctt		
	*P14AS*-S3F	agccugggcuagagacgaatt		
	*P14AS*-S3R	uucgucucuagcccaggcutt		
	*AUF1*-S1F	cguggguucugcuuuauuatt		
	*AUF1*-S1R	uaauaaagcagaacccacgtt		
	*AUF1*-S2F	gccaugucgaaggaacaautt		
	*AUF1*-S2R	auuguuccuucgacauggctt		
	*AUF1*-S3F	cuacuauggauauggugautt		
	*AUF1*-S3R	aucaccauauccauaguagtt		
	Scramble-F	uucuccgaacgugucacgutt		
	Scramble-R	acgugugacacguucggagaatt		

### Plasmid construction and transfection

The 1043-nt *P14AS* lentiviral vector was constructed in PCDH-CMV-EF1a-copGFP-T2A- Puro lentiviral vector by Syngentech Co., Ltd. (Beijing, China). The lentiviruses for the empty control and *P14AS* expression vector were generated with the lentiviral packaging kit (BG20401S, Beijing Syngentech Co., Ltd., China) according to the manufacturer’s manual. Briefly, HEK293FT cells were seeded in 6 cm diameter plates, and transfected with vectors at 40% confluence. The medium was collected after 48 h, and filtered with a 0.45 μm filter. The stably transfected HCT116, SW480 and MGC803 cells were selected for 3 days after infection of these viruses with 1 μg/mL puromycin (Sigma, St. Louis, MO, USA).

The four AUF1 isoforms (p37, p40, p42 and p45 KD) in pcDNA3.1 vector were kindly provided by Professor Xiaotian Zhang at Beijing Normal University [11]. To construct the pGEX-4 T-1-AUF1 expression vectors, the full-length coding region of four AUF1 isoforms was amplified by PCR using the primers (Table 1) and then inserted between the EcoRI and XhoI sites of the pGEX-4 T-1 vector. These proteins were purified with GST-tag from bacteria [12].

### RNA sequencing

The *AUF1* gene was knocked down in stably *P14AS*-overexpressed HCT116 cells for 72 h. The transcriptomes of these cells were sequenced, as described in the supplemental method (Additional file 1). The data sets were deposited in the Gene Expression Omnibus database with the accession number GSE127905. Function annotation for differentially expressed genes was performed using the David 6.8 online tools at the website (https://david.ncifcrf.gov/tools.jsp) [13].

### Knockout of AU-rich element (ARE) in exon 1 of *P14AS* and its promoter by CRISPR/Cas9

A dual gRNA approach was used to knock out the ARE-containing sequence in exon 1 of the *P14AS* gene and its promoter by the CRISPR/Cas9 system, respectively. The oligonucleotides used for sgRNA construction were individually designed upstream and downstream of the target fragment (Table 1) and were synthesized by Thermo Scientific, Inc. (Rockford, IL, USA). The sgRNAs were cloned into the Lenti-CRISPR-V2 vector expressing Cas9 (Plasmid #52961, Addgene, Inc.). Then, the lentiviral plasmid expressing both gRNAs and Cas9 was introduced into HEK293FT cells. The viral supernatants were collected 48 h after transfection and were used to infect HEK293T and HCT116 cells. One week later, the infected cells were subjected to puromycin selection, and surviving cells were seeded into 96-well plates to select the monoclonal cells. Initial identification of knockout cell clones was carried out by genomic PCR, the sequences of out- and in-primers are listed in Table 1. *P14AS* ARE−/promoter-KO-negative clones were pooled and served as a wildtype (WT) control.

### RNA pull-down assay

Biotin-labeled targeted *P14AS* probe #1-#6, and control probe #1-#2 (*Escherichia coli* strain genome) (Table 1) were in vitro synthesized by the Beijing Genomics Institute using PierceTM RNA 3′ End Desthiobiotinylation Kit (20,163, Thermo Scientific, Rockford, IL, USA) These probes were incubated with separated lysates from HEK293T cells, as described in the supplemental methods (Additional file 1).

### RNA immunoprecipitation assay (RIP)

The RIP assay was carried out using the RNA-Binding Protein Immunoprecipitation Kit (Cat# 17–701, EZ-Magna, Millipore, USA) according to the manufacturer’s instructions. Total AUF1-binding RNAs were immunoprecipitated and extracted using AUF1 antibody (ab61193, Abcam, UK). cDNA was synthesized from the RIP-RNAs using random primers, and gene-specific quantitative PCR was then performed using the primer *P14AS*-F1 and -R1 set to amplify the 254-bp fragment within the 1043-nt *P14AS* gene (Table 1).

### Animal experiments

HCT116 cells stably transfected with the control, *P14AS* expression vector or *P14AS* knockout vector were harvested by trypsinization, washed twice with PBS, and then subcutaneously injected into the bilateral inguinal area of female NOD/SCID mice (body weight, 18–20 g; age, 6 weeks, Beijing Huafukang Bioscience Co. Inc., 2 × 10^6^ cells per injection). On the 17th posttransplantation day, mice were sacrificed. For the experiment on tumor growth from *P14AS*^−/−^ and *P14AS*^+/+^ HCT116 cells (2 × 10^6^ cells per injection), the mice were sacrificed on the 19th posttransplantation day. Tumors were fixed in 4% paraformaldehyde, sectioned, and stained with hematoxylin and eosin (H&E).

### Statistical analyses

All statistical analyses were performed using SPSS 18.0 software. The Kolmogorov–Smirnov test was used to estimate the normality of distributions. The Mann–Whitney U-test was conducted for non-normally distributed data. Student’s t-test was conducted for normally distributed data. All statistical tests were two-sided. Statistical significance was assigned at *P* < 0.05 (*) or *P* < 0.01 (**).

### Other methods

Other used methods, including cell culture, Western blotting, RT-PCR, RNA sequencing, RNA-FISH, RNA pull-down, cell proliferation and migration assays, siRNA downregulation, electrophoretic mobility shift assay (EMSA), and induction of methylation of *P16* CpG islands, were listed in the supplemental method (Additional file 1).

## Results

### Characterization of endogenous lncRNA *P14AS* in cancer cells

An RNACap-Seq technology was established and used to screen novel *CDKN2A*-specific RNA transcripts in HEK293T and MCF7 cells (Fig. 1a). A three-exon 1043-nt lncRNA transcribed from the antisense strand of the fragment around exon 1β of the *CDKN2A* locus was detected from the RNACap-Seq readouts (chr9: 21,989,178-21,994,898 in the human genome GRCh37/hg19; (Additional file 2: Fig. S1A). The results of Northern blot analysis confirmed the existence of the endogenous lncRNA *P14AS* in 6 cell lines (BGC823, SGC7901, HepG2, HCT116, HEK293T, and SW480 cells; Fig. 1b). *P14AS* was not detected in the negative control cell lines A549 (without *CDKN2A/B* allele) and AGS (containing fully methylated *P16* alleles).

LncRNAs, including *ANRIL*, are often processed into circular RNAs. To study whether *P14AS* is a circular RNA, the linear RNAs were digested by RNase *R* before cDNA synthesis by reverse transcription (RT). No RT-PCR product was amplified from the RNase *R*-digested samples, indicating that *P14AS* is a linear lncRNA (Fig. 1c). The PhyloCSF analysis results showed that *P14AS* has no coding capacity (Additional file 2: Figure S1B). RNA-FISH analysis revealed that endogenous *P14AS* was mainly distributed in the cytoplasm of HEK293T and SW480 cells (Fig. 1d).

It is well-known that *P14* and *P16* genes are transcribed from different transcription start sites (TSSs) in the human *CDKN2A* locus. Also, *P14* and *P16* mRNAs share the same *CDKN2A* exon 2 with different translation reading frames (Additional file 2: Figure S1a). Similarly, *P14AS* exon 3 (79-nt) completely overlaps with the 5′-sequence in exon 1 of *ANRIL* (Fig. 1a and S1a). Therefore, *ANRIL* and *P14AS* might be spliced from the same primary transcript, and *P14AS* might be an isoform of *ANRIL*. Alternatively, *ANRIL* and *P14AS* may be transcribed from the *CDKN2A* gene using different TSS and partially shared exon 1 of *ANRIL*.

### Direct lncRNA-protein interaction between *P14AS* and AUF1

**Fig. 2:**
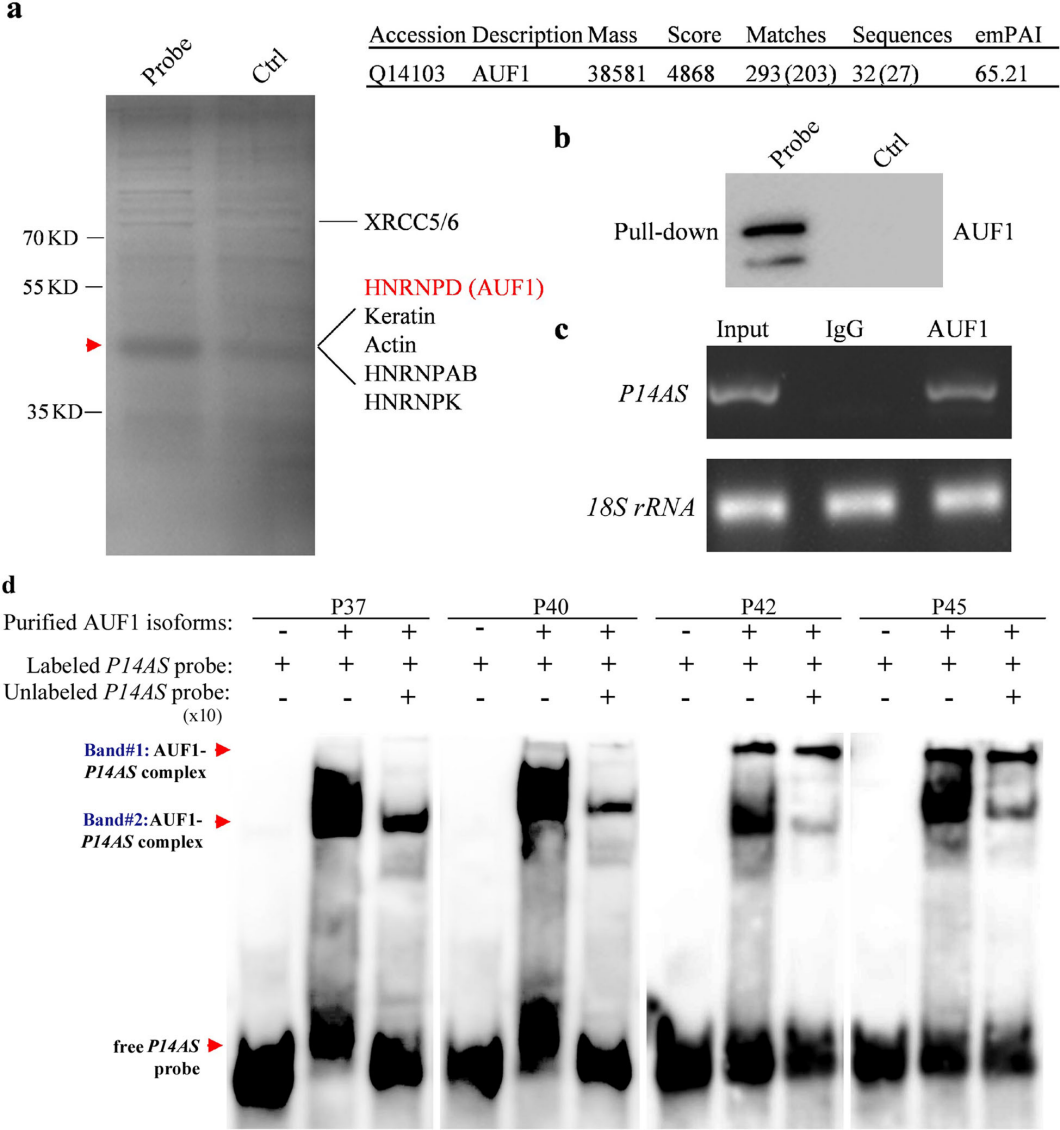
*P14AS* interacts with AUF1 through the AU-rich element in exon 1. **a** Biotin-labeled *P14AS* pull-down complexes from HEK293T cell lysate, following mass spectrometry; (**b**) Western blot analyses; (**c**) Interaction of AUF1 protein with *P14AS* in RIP-PCR; (**d**) Interaction of four purified AUF1 protein isoforms with biotin-labeled *P14AS* probe in the RNA-electrophoretic mobility shift assay (RNA-EMSA). The interaction of Band #2-AUF1 P42 isoform with biotin-labeled *P14AS* probe was almost completely inhibited by the unlabeled probe (×10). However, the interaction of Band #1-AUF1 P42 and P45 isoforms with the *P14AS* was not affected by the unlabeled probe

We performed a biotin-labeled RNA pull-down and mass spectrometry assay to identify potential *P14AS*-binding proteins from HEK293T cell lysates. We found that endogenous AUF1 proteins were the top proteins in the *P14AS* pull-down complexes (Additional file 3: Table S1, Fig. 2a). Western blot analysis confirmed the interaction between AUF1 and *P14AS* (Fig. 2b). To determine whether *P14AS* naturally binds to AUF1 protein in cells (without gene overexpression), we performed the RNA-Immunoprecipitation (RIP) assay and found that *P14AS* was significantly enriched in the AUF1 antibody-RIP complexes compared with the IgG control (Fig. 2c), indicating the occurrence of endogenous *P14AS*-AUF1 binding in cells. The results of the RNA-EMSA assay further confirmed that *P14AS* could directly bind to four AUF1 protein isoforms (Fig. 2d). Unlabeled *P14AS* probe (× 10) could inhibit most of the *P14AS*-binding to AUF1 P37 and P40. In addition, there were two *P14AS*-binding bands for the AUF1 P42 and P45 complexes and unlabeled *P14AS* probe could only inhibit the *P14AS*-binding to one of these AUF1 bands. These results suggest specific bindings between *P14AS* and AUF1, at least for P37 and P40 isoforms.

### *P14AS*-AUF1 interaction upregulates *ANRIL* expression

**Fig. 3:**
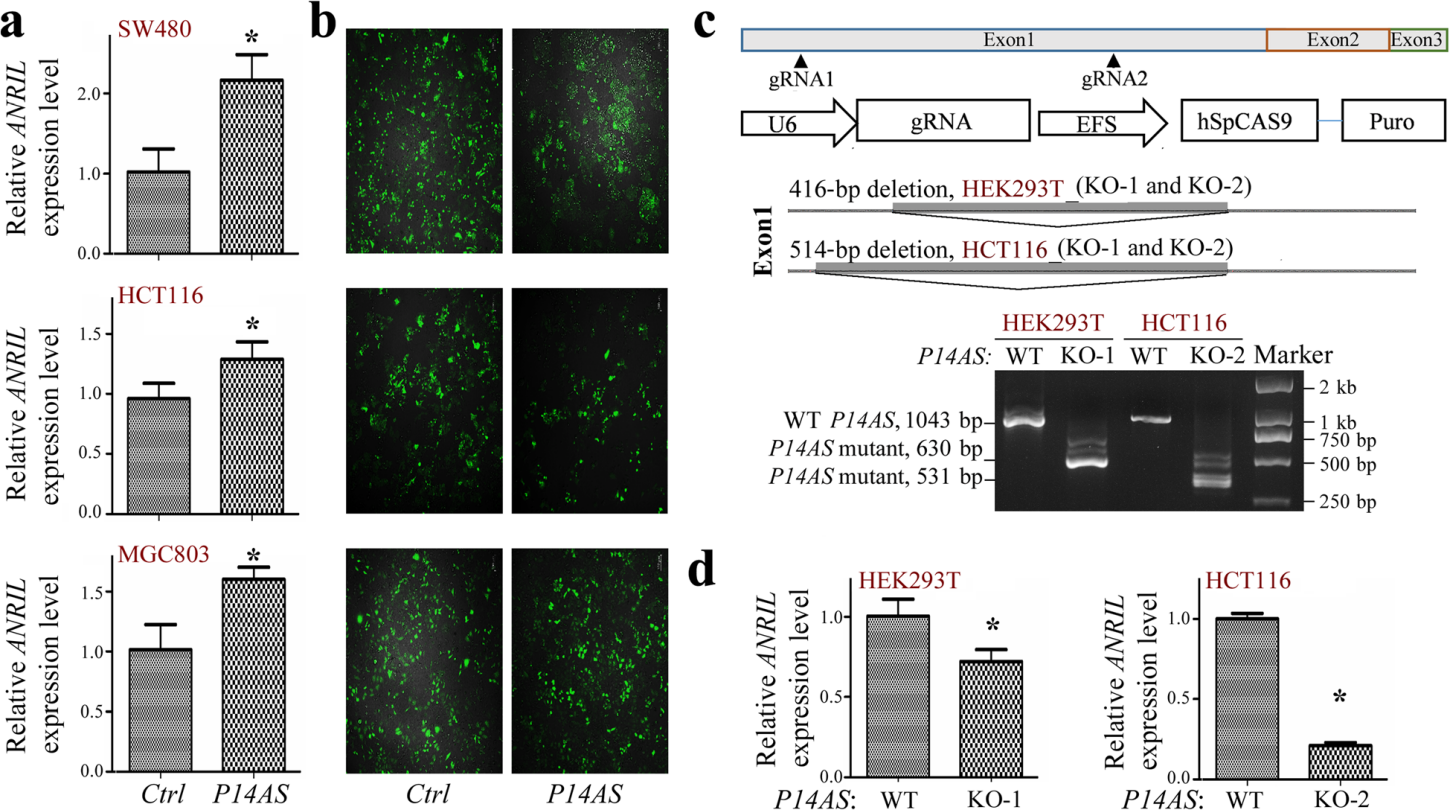
*P14AS* affects the expression level of the *ANRIL* gene. qRT-PCR data are normalized to *GAPDH* mRNA levels and shown as the means ± SD. **a** The expression levels of *ANRIL* in the *P14AS* vector stably transfected colon cancer cells (HCT116 and SW480), and gastric cancer cells (MGC803) were analyzed by qRT-PCR. **b** GFP-expression efficiency in the *P14AS* expression and control vector in the stably transfected cells. **c** Knockout (KO) of the ARE-containing element within *P14AS* exon 1 by CRISPR/Cas9. A 416-bp (chr9: 21,989,309-21,989,724) or 514-bp (chr9: 21,989,211-21,989,724) fragment deletion in *P14AS* exon 1 was detected by RT-PCR (bottom chart) in HEK293T or HCT116 *P14AS*-KO clones. **d** The expression change of *ANRIL* in HCT116 and HEK293T cells whose ARE-containing elements in *P14AS* exon 1 were homogenously deleted in the qRT-PCR analysis. Pooled *P14AS* ARE-KO-negative clones were used as the wild-type (WT) control

As lncRNAs often act as *cis* regulators of nearby genes, we determined whether *P14AS* could affect the expression of *ANRIL* and other neighboring genes. The qRT-PCR results revealed that stable *P14AS* overexpression consistently increased the expression level of *ANRIL* in SW480, HCT116, and MGC803 cells (Fig. 3a-3b). Similar results were also observed for *P16*, *P15*, and *P14* genes hosted by the same 9p21.3 locus (Additional file 4: Figure S2a-S2b).

We then hypothesized that *P14AS*-AUF1 interaction could protect *ANRIL* and other RNAs from degradation. To test this hypothesis, the ARE element in *P14AS* exon 1 (Fig. 1a) was homogenously knocked out (KO) in HCT116 and HEK293T cells using CRISPR/Cas9 technology (Fig. 3c). PCR-sequencing and RT-PCR analyses confirmed the homogenous deletion of *P14AS* exon 1 (416-bp and 514-bp deletions) in the HEK293T (KO-1 and -2) and HCT116 clones (KO-1 and -2) (Additional file 4: Figure S2c). The expression levels of *ANRIL* were significantly decreased in the HCT116 and HEK293T *P14AS*-KO cells in the qRT-PCR analyses (Fig. 3d). Similar decreases of *P15*, *P14*, and *P16* expression levels by *P14AS* knockout were also observed in these clones (Additional file 4: Figure S2c-S2d).

We next determined if *P14AS* impacts *ANRIL*/*P16* RNA stability through interaction with the AUF1 protein. As expected, *ANRIL* and *P16* mRNA indeed existed in the AUF1-RIP RNA complexes in HCT116 cells (Additional file 5: Figure S3a), suggesting that both *ANRIL* and *P16* mRNA are AUF1-binding RNAs. The amount of the AUF1-RIP *ANRIL* and *P16* mRNA was significantly decreased in HCT116 cells with *P14AS* overexpression compared with the control cells (Additional file 5: Figure S3b), suggesting a competitive AUF1-binding between *P14AS* and *ANRIL/P16* RNAs (Additional file 5: Figure S3c).

We further transiently transfected scramble control and *AUF1* siRNA in *P14AS-*overexpressed stable HCT116 cells, and performed RNA sequencing at 72 h after transfection. The results showed that *ANRIL* was one of the *P14AS*-upregulated lncRNAs (fold change > 2.0) while *AUF1* knockdown upregulated many lncRNA genes (*N* = 341). However, after the *AUF1* knockdown, *P14AS* could not upregulate *ANRIL* expression (Additional file 6: Figure S4). In addition, after the *AUF1* knockdown, *P14AS* downregulated many lncRNA genes (*N* = 433). No significant changes in *P15* and *P16* mRNA levels were observed. These results imply that *P14AS* could protect the decay of lncRNAs, including *ANRIL*, in an AUF1-dependent pattern. The results of David functional annotation analyses showed that about half of *P14AS*-upregulated and -downregulated genes were significantly enriched with sequences containing signal peptide feature or related to glycoproteins (Additional file 7: Table S2 and Additional file 8: Table S3). However, 155 of 377 (41%) upregulated genes and 246 of 437 (56%) downregulated genes were not included in the David 6.8 functional annotation analyses. Thus, more functions of *P14AS-*affected RNAs were expected.

Interestingly, a positive relationship between the expression levels of *ANRIL* and *AUF1* mRNA was also observed using the transcript databases for 1037 cell lines in the cancer cell line encyclopedia (CCLE) project [*R* = 0.23 (or 0.30 for 224 cell lines with relative *CDKN2A* copy number > 0), *P* < 0.001; Additional file 9: Figure S5]. Again, the *AUF1* expression did not correlate with that of *P16* and inversely correlated with *P15* expression in these cell lines. Overall, these results suggest that *P14AS* could upregulate the expression of *ANRIL* (and other lncRNAs), probably in an AUF1 binding-dependent pattern.

### Promotion of cell proliferation and tumor formation by *P14AS*

**Fig. 4:**
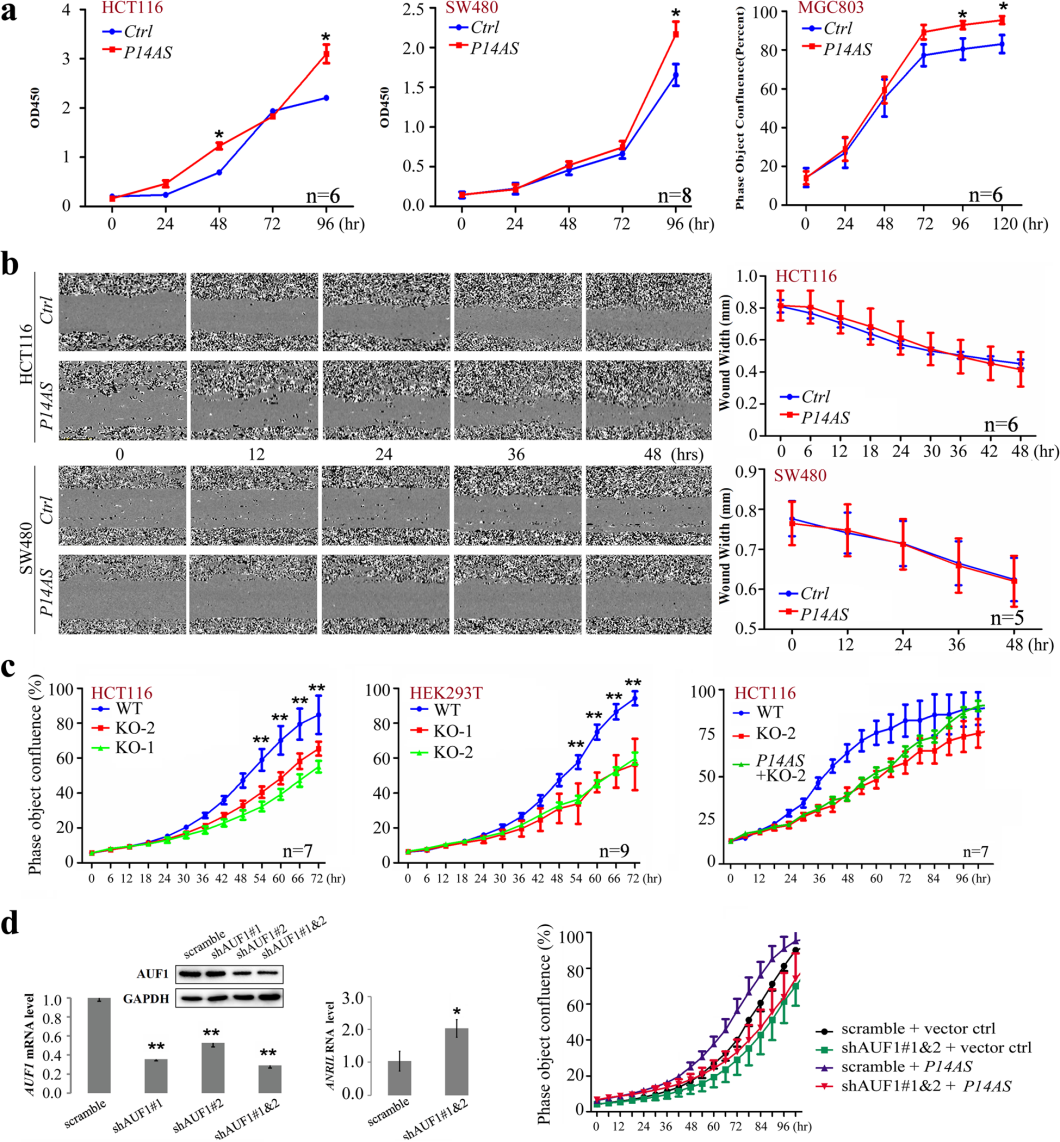
*P14AS* promoted cancer cell proliferation. **a***P14AS* overexpression promoted cell proliferation in the CCK-8 assays and IncuCyte long-term dynamic observation. **b** Wound width curve for HCT116 and SW480 cells with *P14AS* overexpression in the long-term observation. **c** Effect of *P14AS* knockout (KO) by CRISPR/Ccas9 and *P14AS* re-expression in the proliferation of HCT116 and HEK293T cells. **d** Effect of shRNA-knockdown of *AUF1* and/or transient *P14AS* overexpression on the proliferation of HCT116 cells (Right chart). The levels of *AUF1* and *ANRIL* expression in HCT116 cells with stable shAFU1 transfection for four weeks was tested with qRT-PCR (Left and Right charts, respectively) and Western blotting (top chart) before these cells were transiently transfected with the *P14AS* expression vector. Student’s *t*-test: *, *P* < 0.05; **, *P* < 0.01

We further studied the effects of *P14AS* overexpression on cancer cell behavior in vitro and in vivo. Both human colon cell lines SW480 and HCT116 and gastric cancer cell line MGC803 were stably transfected with the *P14AS* expression vector. The results of CCK-8 and long-term dynamic observation assays revealed that *P14AS* overexpression promoted cell proliferation but did not promote cell migration (Fig. 4a and b).

Moreover, the proliferation of both HCT116 and HEK293T cells was significantly inhibited by the *P14AS*-KO compared to the wild-type (WT) controls (Fig. 4c, left and middle, respectively). The results of rescue experiments showed that enforced *P14AS* re-expression could completely recover the *P14AS*-KO-inhibited proliferation of HCT116 cells after 96 h, though the proliferation difference was still observed at the beginning between the *P14AS*-KO cells and *P14AS* re-expressing cells after cell seeding (Fig. 4c, right). Notably, stably *AUF1* shRNA-knockdown completely abolished the *P14AS-*induced enhancement of the proliferation of HCT116 cells (Fig. 4d). As expected, the level of *ANRIL* expression was significantly increased in these cells with stably *AUF1* knockdown. Since the *P16* gene is inactivated in HCT116 cells, endogenous lncRNA *P14AS* may serve as a sponge to protect other AUF1 targets from decay and promote cancer cell proliferation. These phenomena suggest that the enhancement of cell proliferation by *P14AS* may depend on the ARE element in *P14AS* exon 1.

**Fig. 5:**
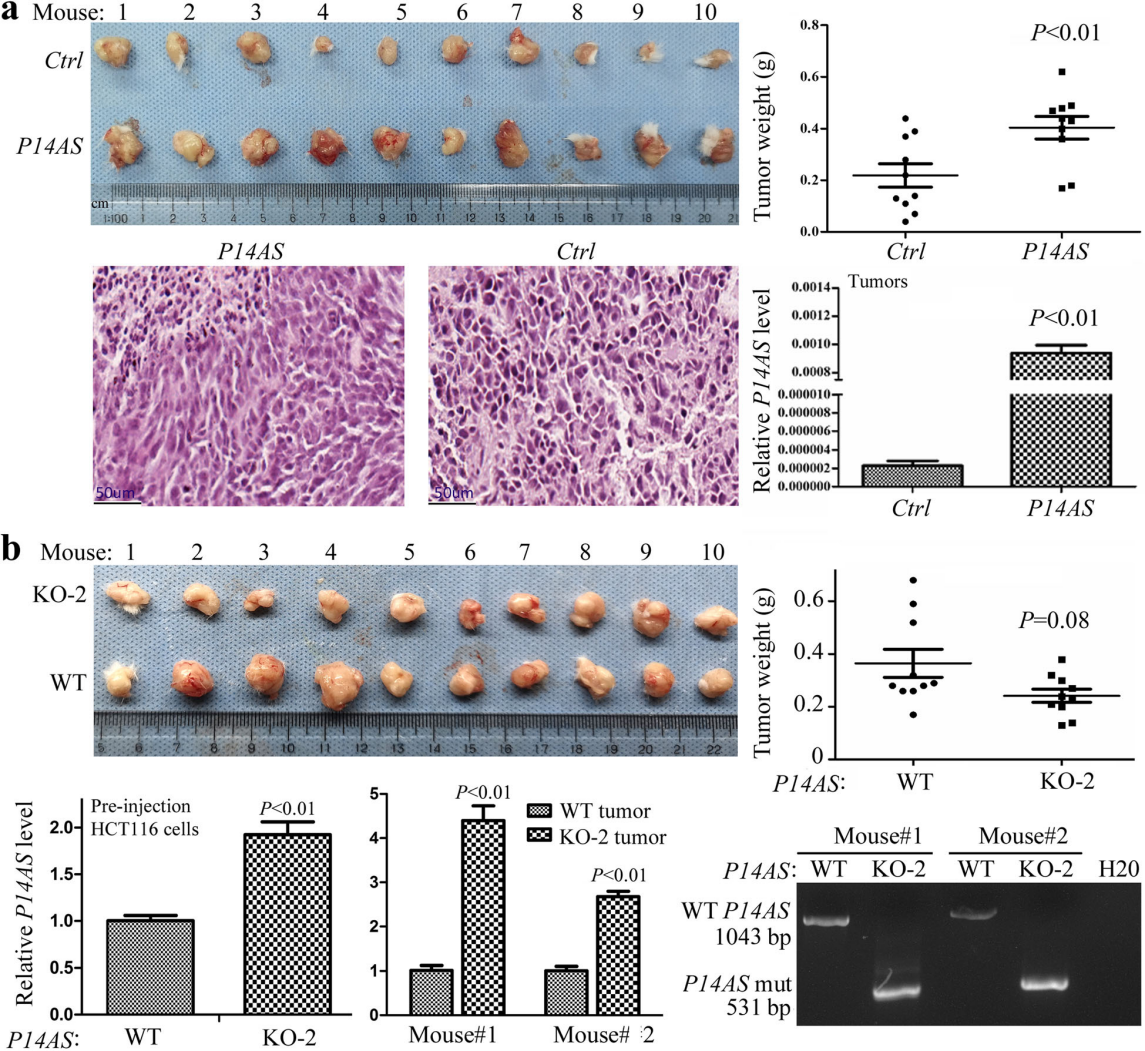
Effect of *P14AS* overexpression and knockout on tumor growth from HCT116 cells in NOD SCID mice. (**a**) Tumors derived from HCT116 cells stably transfected with the *P14AS* and control vectors on the 17th posttransplantation day. Hematoxylin and eosin (H&E) staining images were attached. (**b**) Tumors derived from *P14AS-*knockout HCT116 KO-2 clone on the 19th posttransplantation day. The tumor weight and the levels of wild-type *P14AS* (WT) and its KO-truncated counterpart were displayed. Ten mice per group

To validate the in vitro results, NOD-SCID mice (*N* = 10) were injected with HCT116 cells stably transfected with *P14AS* into the left inguinal area and the empty control vector into the right inguinal area for each mouse. On the 17th posttransplantation day, the number and weight of tumors in the *P14AS* group were significantly higher than those in the control group (*P* < 0.01, Fig. 5a). The qRT-PCR results indicate that *P14AS* remained to be actively transcribed in these tumors. In contrast, knockout of the ARE-containing element in *P14AS* exon 1 decreased the growth of HCT116 cells (*P* = 0.08, Fig. 5b). The qRT-PCR results illustrated that the level of KO-truncated *P14AS* lncRNA was higher in the *P14AS*-KO cells than that of wild-type *P14AS* in the pre-injection *P14AS*-KO cells and derived tumors. Thus, knockout of the exon 1 ARE-containing element may lead to compensatory upregulation of the truncated *P14AS* gene residue. Collectively, these results indicate that *P14AS* promotes cancer cell proliferation in vitro and tumor formation in vivo.

### Coordinate overexpression of *P14AS, ANRIL,* and *AUF1* in colon cancer tissues

To determine whether *P14AS*, *ANRIL*, and *P16* expression were coordinately upregulated in cancer development, we examined their expression status in colon cancer (CC) and their corresponding surgical margin (SM) tissue samples from 172 patients, and normal colon mucosa biopsy samples from 50 noncancer patients. The results of qRT-PCR detection (254-bp) revealed that the average *P14AS* level in CC tissues, with and without *ANRIL* expression, was significantly higher than that in the paired SM tissues and normal biopsies (Additional file 10: Figure S6a-b). The average *P14AS* level in SM samples was also markedly higher than that in normal colon biopsies. More *P14AS* was detected in CCs with advanced local invasion (trend-test, *P* = 0.034; Additional file 11: Table S4). These phenomena suggest that *P14AS* upregulation is an early event in CC development and correlates with CC invasion.

In addition, the positive rate of *ANRIL* (by RT-PCR) was significantly higher in CCs than SMs (90/167 [53.9%] vs. 52/167 [31.1%], *P* < 0.001; Additional file 13: Table S5). Similarly, the average *P16* mRNA level was also significantly higher in CCs than SMs (Additional file 10: Figure S6a). In contrast, the average *P15* mRNA level in CCs was considerably lower than that in SM tissues. The level of *AUF1* mRNA (by qRT-PCR) was significantly higher in CCs than SMs (*median*, 22.02 vs. 1.77, *P* < 0.001; Additional file 14: Table S6). While no significant assoication between clinicopathological characteristics and the level of *AUF1* mRNA was detected for CCs, the level of *AUF1* mRNA was higher for SMs from patients with stage I&II CC than those with stage III&IV (Mann–Whitney U-test, *P* = 0.014).

The *P16* mRNA level (by qRT-PCR) was also significantly higher in the *P14AS*-positive colon CC and SM tissues (by RT-PCR; 1043-bp) than in the *P14AS*-negative tissues (nonparametric tests, *P* = 0.039; Additional file 10: Figure S6c). Once again, no significant correlations were observed between the levels of *P14AS* and *P15* mRNA (Additional file 10: Figure S6d). Thus, *P14AS* expression is coordinately overexpressed with *ANRIL* and *P16* in colon tissues.

In addition, the level of *AUF1* mRNA was positively associated with *P14AS* expression in CC or SM samples (Pearson_r = 0.27, *P* = 0.002; Additional file 12: Figure S7a). The level of *AUF1* mRNA was significantly higher in *ANRIL*-positive SMs than *ANRIL*-negative SMs (*P* = 0.047). A similar difference was also observed between CCs with and without *ANRIL* expression (*P* = 0.061; Additional file 12: Figure S7b).

Combined analysis showed that the frequency of P14AS and ANRIL coexpression was significantly higher in distant metastatic colon cancers than non-metastatic colon cancers (53.3% vs. 30.7%, *P* = 0.018; Additional file 15: Table S7). Such an association could not be observed when P14AS and ANRIL were individually analyzed.

### Characterization of the *P14AS* promoter

**Fig. 6:**
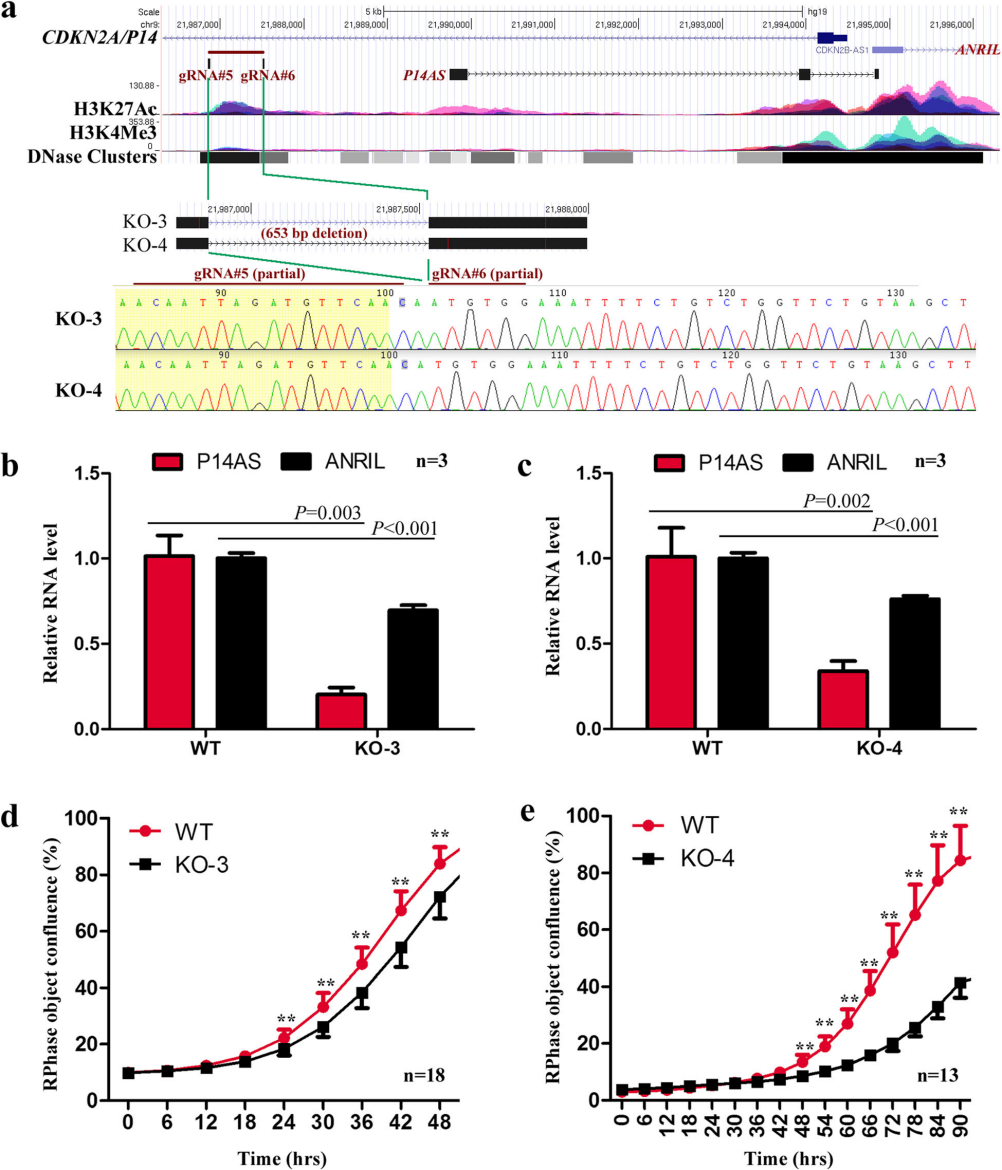
Effects of the knockout of the *P14AS* promoter on the *P14AS* and *ANRIL* transcription and the proliferation of HEK293T cells. (**a**) Remaining sequences of the *P14AS* promoter with a 653 bp deletion (chr9:21,986,875-21,987,527; hg19) by CRISPR/Cas9 in two HEK293T subclones (KO-3 and KO-4). Locations of the wildtype *P14AS* promoter, two guide RNAs, and the 653 bp knockout (KO) fragment are also illustrated; (**b** and **c**) The levels of *P14AS* and *ANRIL* expression in wildtype and KO-3 or KO-4 cells by qRT-PCR. (**d** and **e**) Effect of the promoter deletion on KO-3 and KO-4 cell proliferation in the IncuCyte long-term dynamic observation. Pooled *P14AS* promoter-KO-negative clones were used as a wild-type (WT) control. Error bars, S.D. **, *P* < 0.01

According to the ENCODE datasets for 7 cell lines, there is a promoter-like sequence 2-kb upstream of *P14AS* exon 1. This region is enriched with active H3K27Ac and H3K4Me3 modifications (Additional file 16: Figure S8a-S8b), where RNA polymerase II (POLR2A)-binding was detected (Additional file 16: Figure S8c, highlighted in red lines). This promoter-like sequence is conserved among vertebrates (Additional file 16: Figure S8d) and implies its biological significance. To study whether this sequence is the *P14AS* promoter, we knocked out of this sequence (653-bp) with CRISPR/Cas9 and found that the level of *P14AS* transcription was dramatically decreased in two HEK293T subclones (KO-3 and KO-4; Fig. 6a-c). In addition, the level of *ANRIL* expression (by qRT-PCR) was significantly reduced (Fig. 6d). The results of long-term dynamic observation assays showed that the proliferation of these *P14AS* promoter-KO cells was also repressed (Fig. 6e). These data strongly suggest that this sequence may be the *P14AS* promoter*.*

## Discussion

Accumulating evidence has revealed that lncRNAs function in multiple cellular processes, including transcription, intracellular trafficking, chromosome remodeling, and disease development. Deregulation of lncRNAs was found in various cancers [14–16]. The 9p21 locus is the most frequently deleted allele in cancer genomes. This locus contains several important tumor suppressor genes (*CDKN2A/B* encodes the P16, P14, and P15 proteins). It also hosts the oncogenic antisense lncRNA gene *ANRIL*, which is implicated in cancers of the colon, breast, lung, and bladder [17–22]. In the present study, we characterized a new oncogenic antisense lncRNA, *P14AS*, in this locus. Our systemic studies show that *P14AS cis* upregulates *ANRIL* and promotes cancer cell proliferation.

We found that *P14AS* was frequently overexpressed in CC tissues, and that *P14AS* overexpression promoted the proliferation of cancer cells. In contrast, *P14AS* knockdown by siRNA and knockout of the ARE-containing element in *P14AS* exon 1 by CRISPR/Cas9 could repress cancer cell proliferation. The effect of *P14AS* on cancer cell proliferation was confirmed in a mouse model. These results suggest that *P14AS* may be an oncogene. We also analyzed the expression status of *P14AS* in gastric carcinoma tissues (*N* = 40), *P14AS* expression was not detected (data not shown). This result warrants studying its roles in cancer development in other organs.

In addition, although *P14AS* could upregulate *P16* expression in cell lines and *P14AS* expression is positively correlated with *P16* expression in colon tissues, *P14AS* overexpression or knockout remains to affect the proliferation of the *P16*-inactive cell lines (HCT116 and SW480), suggesting that *P14AS* may function as an oncogene in a *P16*-independent way. P16 is a weakly expressed nucleic protein in normal cells. However, the P16 protein is sequestered in the cytoplasm without tumor suppressor function in many cancer tissues, including CCs [6]. Therefore, the upregulation of *P16* expression by *P14AS* in cancers may not affect cancer development. Instead, dysfunctions of *P16*, *P14*, and *P15* cause abnormal G1-S shift in the cell cycle that plays crucial role in the malignant transformation of human cells due to increased phosphorylation of RB1 [2].

AUF1 is a member of the family of RNA binding proteins that is termed ARE/poly (U)-binding/ degradation factor 1. The canonical functions of AUF1 are to regulate mRNA decay and translation via recognition of specific sequence elements in mRNA 3′ untranslated regions (3’UTR) [23]. For example, AUF1 is involved in the regulation of *c-MYC* and *P16* mRNA stability [7, 8]. Previous studies have identified AUF1 as an important destabilizer for *P16* mRNA, thereby influencing cell senescence [24]. Moreover, AUF1 can interact with lncRNAs, including *NEAT1* (10) and *linc-ROR* [9]. Interestingly, we found that there is an ARE element in *P14AS* exon 1 that binds to AUF1. Notably, knockout of the ARE-containing element not only abolishes the function of *P14AS* to upregulate *ANRIL* and *P16/P14/P15* expression but also eliminates the effect of *P14AS* on cancer cell proliferation. Because re-expression of the wild-type *P14AS* could restore the effect of *P14AS*-KO on cell proliferation and stable knockdown of *AUF1* expression could abolish the effect of *P14AS* on cell proliferation, *P14AS*-AUF1 binding is highly considered to be the main pathway for the function of *P14AS*.

It has been reported that AUF1 isoforms (P37, P40, P42, And P45) could form dimers, which could yield oligomeric AUF1 complexes [25, 26]. In our RNA-EMSA assay, most *P14AS-*binding to the AUF1 P37 and P40 band was inhibited by the unlabeled *P14AS* probe (× 10), suggesting high binding specificity between these AUF1 isoforms to *P14AS*.

Moreover, we found that *P14AS* overexpression and knockout/knockdown significantly increased and decreased *ANRIL* expression in many cell lines, respectively. The results of our RNA-seq analyses confirmed the upregulation (fold change > 2.5) of *ANRIL* expression by *P14AS* in HCT116 cells. Both *P14AS* and *ANRIL* were upregulated in CC tissues, and more *P14AS* was detected in the *ANRIL*-positive tissues than the *ANRIL*-negative tissues. In addition, the results of our AUF1 RIP-PCR experiment indicate that *ANRIL* lncRNA is an AUF1-binding RNA. Analysis of the public CCLE data reveals that the *AUF1* mRNA level was positively and significantly correlated with the *ANRIL* lncRNA level in 1037 human cancer cell lines. The level of *AUF1* mRNA was positively and significantly associated with the *P14AS* level in CC tissues. These findings support that *P14AS* may function as an oncogene through the *cis* upregulation of *ANRIL* expression in cancer cells through AUF1*-P14AS* binding.

Consistent with the reported functions of AUF1 in degrading RNAs, AUF1 knockdown by siRNA could upregulate the expression of many lncRNAs in HCT116 cells in our RNA-seq analysis. However, in cells with *AUF1* knockdown, *P14AS* downregulated the expression level of many lncRNAs (fold change <− 2.0), which was not observed in cells with *P14AS* overexpression alone. This implies that AUF1 may be a dominant factor in regulating *P14AS* functions.

It is well-recognized that DNA demethylation of CpG islands around TSS is needed for gene transcription [27] and that methylation of CpG islands in the gene body is needed for active genes to repress abnormal transcription from cryptic TSSs [28]. Surprisingly, it was reported that the *ANRIL* expression level was positively associated with the methylation level of the *P14* promoter CpG islands in tissues [29], implying that the *P14* promoter CpG islands may not be the true *ANRIL* promoter but the *ANRIL* gene body CpG islands. We also reported that *ANRIL* and *P16* are coordinately transcribed in CCLE cell lines, and *P16* methylation repressed both *ANRIL* and *P16* expression and did not affect *P14/P15* expression [6]. Furthermore, we found that *P16* methylation repressed *P14AS* expression in BGC 823 cells (Additional file 17: Figure S9), suggesting a co-repression of *P14AS*, *ANRIL*, and *P16* expression by DNA methylation of the *P16* CpG island around TSS. Chromosome conformation capture (3C) analysis showed that the *P16* promoter could play a key role in formation of compact chromatin loops [30]. *P16* promoter methylation may lead to focal chromatin condensing and downregulation of these genes. We also found that the 79-nt sequence of *P14AS* exon 3 was completely overlapped with the 5′-sequence of *ANRIL* exon 1. To test the probability that *P14AS* and *ANRIL* might be spliced from the same primary transcript, we knocked out the promoter-like sequence 2-kb upstream of *P14AS* exon 1 with CRISPR/Cas9 and found a dramatically decreased *P14AS* transcription and slightly decreased *ANRIL* expression in two subclones, providing strong evidence to support the conserved sequence is the *P14AS* promoter. The fact that KO of the *P14AS* promoter only weakly decreased *ANRIL* transcription suggests that the *P14AS* promoter is likely not the *ANRIL* promoter or the *ANRIL* gene could be transcribed using multiple TSSs.

Unlike mature mRNAs that may contain many exons, approximately 42% of mature lncRNAs contain only two exons [31]. In melanoma cells, preliminary *ANRIL* transcripts could be spliced into diverse mature forms, including linear *ANRILs* mainly located in the nucleus and circular *ANRILs* located in the cytoplasm, which imply function difference between linear and circular *ANRILs* [32]. The specific processing patterns for lncRNAs make the characterization of lncRNA genes much more complicated than the identification of mRNAs. This processing might account for misidentification of TSSs for lncRNA genes.

It was reported that *ANRIL* knockdown inhibited the proliferation migration of hepatoma cells [33, 34]. However, we found that *P14AS* overexpression or knockout only increased or decreased the proliferation of cell lines HCT116, SW480, and HEK293T, but did not affect the migration of these cells. As we observed in RNA-sequencing data, *P14AS* could target to both *ANRIL* and other ncRNAs in AUF1-dependent manner. This may account for the difference of biological functions between *ANRIL* and *P14AS.* It is worth studying whether linear or circular *ANRIL* transcription and degradation are regulated by *P14AS*.

## Conclusions

The antisense strand of the *CDKN2A* locus hosts the *P14AS* gene, which might be connected with a true TSS for the *ANRIL* gene*. P14AS* is an AUF1-binding lncRNA that upregulates the expression of AUF1 target genes, including *ANRIL*. *P14AS* maybe an oncogenic lncRNA involved in the development of colon cancer.

## Supplementary information

**Additional file 1.** Supplementary methods

**Additional file 2 Figure S1.** Characterization of *P14AS* in the *CDKN2A/B* locus. (**A**) Sashimi view for transcripts detected by CDKN2A-specific probe-captured RNA (RNACap)-Seq in HEK293T cells containing two wild-type CDKN2A/B alleles and in MCF7 cells with the homogenous *P16* deletion. (**B**) Graphic view of the protein-coding potential for *P14AS*, *CDKN2A/P14*, and *ANRIL* genes (adapted from the UCSC website).

**Additional file 3 Table S1**. List of proteins interacted with P14AS characterized in the HEK293T cells in RNA pull-down mass spectrum analysis

**Additional file 4 Figure S2.***P14AS* affects the expression level of the neighboring genes at 9p21.3. qRT-PCR data are normalized to *GAPDH* mRNA levels and shown as the means ± SD. (**a**) The expression levels of *P14*, *P16*, and *P15* genes in the *P14AS* vector stably transfected colon cancer cells (HCT116 and SW480), and gastric cancer cells (MGC803) were analyzed by qRT-PCR. (**b**) Detection of P16/P15/P14 proteins in MGC803 cells in Western blot analyses. (**c**) A fragment deletion in *P14AS* exon 1 was detected by PCR (top chart) in HEK293T or HCT116 *P14AS*-KO clones (KO). The expression changes of *P14*, *P16*, and *P15* genes in HCT116 and HEK293T cells whose ARE-containing elements in *P14AS* exon-1 were homogenously deleted in the qRT-PCR analysis (bottom chart). Pooled *P14AS* ARE-KO-negative subclones were used as a wild-type (WT) control. (**d**) Detection of the P16, P15, and P14 proteins in HEK293T cells in Western blot analyses.

**Additional file 5 Figure S3**. *P14AS* expression decreased *P16* mRNA-AUF1 binding. (**a**) AUF1 directly bound to *ANRIL* and *P16* mRNA in HCT116 cells in the AUF1-RIP-PCR. (**b**) *P14AS* overexpression decreased *ANRIL* and *P16* mRNA-AUF1 interaction in by the AUF1-RIP-qPCR. (**c**) An illustration of how the competitive AUF1-*P14AS* binding protects *ANRIL* and *P16* mRNA from the decay. AUF1 complexes were drawn as dimers based on the reports that AUF1 isoforms (p37, p40, p42, and p45) could form functional dimers [25, 26].

**Additional file 6 Figure S4.** Genome-wide analyses of transcriptome by RNA sequencing for HCT116 cells with and without *P14AS* overexpression and/or AUF1 downregulation. The HCT116 cells with stable *P14AS* overexpression were transfected with *AUF1* siRNAs (siAUF1) for 72 h, and then harvested for RNA sequencing. The number of genes with > 2 fold changes (UP, upregulated; Down, downregulated) for different types of RNAs were labeled. Western blot analysis for the determination of *AUF1* downregulation by siRNAs was inserted into the top chart. Two samples were sequenced for each group.

**Additional file 7 Table S2**. Function annotations for *P14AS*-upregulated genes (*n* = 241) with fold change > 2 in HCT116 cells with the David 6.8: Functional Annotation Tools at the website http://david.ncifcrf.gov/tools.jsp [13]

**Additional file 8 Table S3**. Function annotations for *P14AS*-downregulated genes (*n* = 299) with fold change > 2 in HCT116 cells with the David 6.8: Functional Annotation Tools at the website http://david.ncifcrf.gov/tools.jsp [13]

**Additional file 9 Figure S5.** Association analyses between the expression levels of *AUF1* and *ANRIL*, *P16*, or *P15* using the publicly available transcriptome databases for human cancer cell lines in the CCLE project. (**A**) All 1037 cell lines; (**B**) 224 cell lines without the *CDKN2A* allele deletion (relative copy number > 0).

**Additional file 10 Figure S6.** Comparison of the levels of *P14AS*, *P16*, and *P15* expression in colon tissues from cancer and noncancer patients. (**a**) The expression status of *P14AS*, *P16*, and *P15* in colon cancer (CC), paired surgical margin (SM), and normal colon biopsy (Normal) tissues from noncancer patients by qRT-PCR. (**b**) The level of *P14AS* expression in *ANRIL*-positive and -negative (by RT-PCR) colon CC and SM tissues. (**c** and **d**) Comparison of the expression levels of *P16* and *P15* mRNA (by qRT-PCR) in *P14AS*-positive and -negative (by RT-PCR; 1043 bp) colon CC and SM tissues. Error bars, S.E.M. *, *P* < 0.05; **, *P* < 0.01; N.S. no significance.

**Additional file 11 Table S4**. Comparison of the *P14AS* expression level (by qRT-PCR) in colon cancer (CC) and surgical margin (SM) tissue samples from patients with different clinicopathological characteristics

**Additional file 12 Figure S7.** Comparisons of the levels of *AUF1* mRNA (by qRT-PCR) with those of *P14AS* and *ANRIL* lncRNA in colon cancer tissues (CCs).

**Additional file 13 Table S5**. Comparison of the *ANRIL* expression level (by RT-PCR) in colon cancer (CC) and surgical margin (SM) tissue samples from patients with different clinicopathological characteristics

**Additional file 14 Table S6**. Comparison of the *AUF1* mRNA level (by qRT-PCR) in colon cancer (CC) and surgical margin (SM) tissue samples from patients with different clinicopathological characteristics

**Additional file 15 Table S7**. Comparison of *P14AS* and *ANRIL* coexpression in colon cancer (CC) and surgical margin (SM) tissue samples from patients with different clinicopathological characteristics

**Additional file 16 Figure S8.** Graph of the *P14AS* gene in the *CDKN2A/B* locus. (**A**) CpG islands within the *CDKN2A/P14* gene. (**B**) The transcription and active histone modification status in the chromatin upstream of the *P14AS* gene in 7 cell lines from ENCODE. (**C**) Transcription factors binding to various fragments around the *P14AS* gene from ENCODE. The RNA polymerase II (POLR2A) is highlighted in red lines. (**D**) The conservation status of various fragments among vertebrates (adapted from the UCSC website).

**Additional file 17 Figure S9.** Repression of *P16*, *P14AS*, and *ANRIL* expression in gastric cancer cells by engineered *P16*-specific DNA methyltransferase (P16-Dnmt). The gene expression level was detected using qRT-PCR assay. The detailed processes for the construction of P16-Dnmt and transfection experiments are previously described [27]

## Data Availability

The data and materials of the study are available from the corresponding author upon reasonable request.

## Abbreviations

ARE: AU-rich element
CC: colon cancer
KO: knockout
lncRNA: long non-coding RNA
P16-Dnmt: engineered *P16-*specific zinc-protein-based DNA methyltransferease
qRT-PCR: quantitative reverse transcription-PCR
RIP: RNA immunoprecipitation
RNACap-Seq: probe captured-RNA extra-deep sequencing
RNA-EMSA: RNA-electrophoretic mobility shift assay
SM: surgical margin

## References

[1] Serrano M, Hannon GJ, Beach D (1993). A new regulatory motif in cell-cycle control causing specific inhibition of cyclin D/CDK4. Nature.

[2] Lukas J, Parry D, Aagaard L, Mann DJ, Bartkova J, Strauss M, Peters G, Bartek J (1995). Retinoblastoma-protein-dependent cell-cycle inhibition by the tumour suppressor p16. Nature.

[3] Pasmant E, Laurendeau I, Héron D, Vidaud M, Vidaud D, Bièche I (2007). Characterization of a germ-line deletion, including the entire INK4/ARF locus, in a melanoma-neural system tumor family: identification of ANRIL, an antisense noncoding RNA whose expression coclusters with ARF. Cancer Res.

[4] Yu W, Gius D, Onyango P, Muldoon-Jacobs K, Karp J, Feinberg A, Cui H (2008). Epigenetic silencing of tumour suppressor gene p15 by its antisense RNA. Nature.

[5] Montes M, Nielsen MM, Maglieri G, Jacobsen A, Højfeldt J, Agrawal-Singh S, Hansen K, Helin K, van de Werken HJG, Pedersen JS, Lund AH (2015). The lncRNA MIR31HG regulates p16(INK4A) expression to modulate senescence. Nat Commun.

[6] Gan Y, Ma W, Wang X, Qiao J, Zhang B, Cui C, Liu Z, Deng D (2018). Coordinate transcription of A*NRIL* and P*16* genes silenced by DNA methylation. Chin J Cancer Res.

[7] Jones TR, Cole MD (1987). Rapid cytoplasmic turnover of c-myc mRNA: requirement of the 3′ untranslated sequences. Mol Cell Biol.

[8] Wang W, Martindale JL, Yang X, Chrest FJ, Gorospe M (2005). Increased stability of the p16 mRNA with replicative senescence. EMBO Rep.

[9] Huang J, Zhang A, Ho TT, Zhang Z, Zhou N, Ding X, Zhang X, Xu M, Mo YY (2016). Linc-RoR promotes c-Myc expression through hnRNP I and AUF1. Nucleic Acids Res.

[10] Yoon JH, De S, Srikantan S, Abdelmohsen K, Grammatikakis I, Kim J, Kim KM, Noh JH, White EJ, Martindale JL (2014). PAR-CLIP analysis uncovers AUF1 impact on target RNA fate and genome integrity. Nat Commun.

[11] Sun S, Zhang X, Lyu L, Li X, Yao S, Zhang J (2016). Autotaxin expression is regulated at the post-transcriptional level by the RNA-binding proteins HuR and AUF1. J Biol Chem.

[12] Schäfer F, Seip N, Maertens B, Block H, Kubicek J (2015). Purification of GST-tagged proteins. Methods Enzymol.

[13] Huang Da Wei, Sherman Brad T, Lempicki Richard A (2008). Systematic and integrative analysis of large gene lists using DAVID bioinformatics resources. Nature Protocols.

[14] Bonasio R, Shiekhattar R (2014). Regulation of transcription by long noncoding RNAs. Annu Rev Genet.

[15] Schmitt AM, Chang HY (2016). Long noncoding RNAs in Cancer pathways. Cancer Cell.

[16] Bolha L, Ravnik-Glavač M, Glavač D (2017). Long noncoding RNAs as biomarkers in Cancer. Dis Markers.

[17] Khorshidi HR, Taheri M, Noroozi R, Sarrafzadeh S, Sayad A, Ghafouri-Fard S (2017). Genetic variants in Iranian breast Cancer patients. Cell J.

[18] Meseure D, Vacher S, Alsibai KD, Nicolas A, Chemlali W, Caly M, Lidereau R, Pasmant E, Callens C, Bieche I (2016). Expression of ANRIL-Polycomb complexes-CDKN2A/B/ARF genes in breast tumors: identification of a two-gene (EZH2/CBX7) signature with independent prognostic value. Mol Cancer Res.

[19] Naemura M, Tsunoda T, Inoue Y, Okamoto H, Shirasawa S, Kotake Y (2016). ANRIL regulates the proliferation of human colorectal cancer cells in both two- and three-dimensional culture. Mol Cell Biochem.

[20] Nie FQ, Sun M, Yang JS, Xie M, Xu TP, Xia R, Liu YW, Liu XH, Zhang EB, Lu KH, Shu YQ (2015). Long noncoding RNA ANRIL promotes non-small cell lung cancer cell proliferation and inhibits apoptosis by silencing KLF2 and P21 expression. Mol Cancer Ther.

[21] Sun Z, Ou C, Ren W, Xie X, Li X, Li G (2016). Downregulation of long non-coding RNA ANRIL suppresses lymphangiogenesis and lymphatic metastasis in colorectal cancer. Oncotarget.

[22] Zhu H, Li X, Song Y, Zhang P, Xiao Y, Xing Y (2015). Long non-coding RNA ANRIL is up-regulated in bladder cancer and regulates bladder cancer cell proliferation and apoptosis through the intrinsic pathway. Biochem Biophys Res Commun.

[23] White Elizabeth J.F., Matsangos Aerielle E., Wilson Gerald M. (2016). AUF1 regulation of coding and noncoding RNA. Wiley Interdisciplinary Reviews: RNA.

[24] Chang N, Yi J, Guo G, Liu X, Shang Y, Tong T, Cui Q, Zhan M, Gorospe M, Wang W (2010). HuR uses AUF1 as a cofactor to promote p16INK4 mRNA decay. Mol Cell Biol.

[25] Wilson GM, Sun Y, Lu H, Brewer G (1999). Assembly of AUF1 oligomers on U-rich RNA targets by sequential dimer association. J Biol Chem.

[26] Zucconi BE, Ballin JD, Brewer BY, Ross CR, Huang J, Toth EA, Wilson GM (2010). Alternatively expressed domains of AU-rich element RNA-binding protein 1 (AUF1) regulate RNA-binding affinity, RNA-induced protein oligomerization, and the local conformation of bound RNA ligands. J Biol Chem.

[27] Cui C, Gan Y, Gu L, Wilson J, Liu Z, Zhang B, Deng D (2015). P16-specific DNA methylation by engineered zinc finger methyltransferase inactivates gene transcription and promotes cancer metastasis. Genome Biol.

[28] Brocks D, Schmidt CR, Daskalakis M, Jang HS, Shah NM, Li D, Li J, Zhang B, Hou Y, Laudato S (2017). DNMT and HDAC inhibitors induce cryptic transcription start sites encoded in long terminal repeats. Nat Genet.

[29] Lillycrop K, Murray R, Cheong C, Teh AL, Clarke-Harris R, Barton S, Costello P, Garratt E, Cook E, Titcombe P (2017). ANRIL promoter DNA methylation: a perinatal marker for later adiposity. EBioMedicine.

[30] Hirosue A, Ishihara K, Tokunaga K, Watanabe T, Saitoh N, Nakamoto M, Chandra T, Narita M, Shinohara M, Nakao M (2012). Quantitative assessment of higher-order chromatin structure of the *INK4/ARF* locus in human senescent cells. Aging Cell.

[31] Derrien T, Johnson R, Bussotti G, Tanzer A, Djebali S, Tilgner H, Guernec G, Martin D, Merkel A, Knowles DG (2012). The GENCODE v7 catalog of human long noncoding RNAs: analysis of their gene structure, evolution, and expression. Genome Res.

[32] Sarkar D, Oghabian A, Bodiyabadu PK, Joseph WR, Leung EY, Finlay GJ, Baguley BC, Askarian-Amiri ME (2017). Multiple isoforms of ANRIL in melanoma cells: structural complexity suggests variations in processing. Int J Mol Sci.

[33] Huang Y, Xiang B, Liu Y, Wang Y, Kan H (2018). LncRNA CDKN2B-AS1 promotes tumor growth and metastasis of human hepatocellular carcinoma by targeting let-7c-5p/NAP1L1 axis. Cancer Lett.

[34] Huang D, Bi C, Zhao Q, Ding X, Bian C, Wang H, Wang T, Liu H (2018). Knockdown long non-coding RNA ANRIL inhibits proliferation, migration and invasion of HepG2 cells by down-regulation of miR-191. BMC Cancer.

