# Supplementary material for: Characterization of novel LncRNA P14AS as a protector of ANRIL through AUF1 binding in human cells
Source: Mol Cancer. 2020 Feb 27;19:42. doi: 10.1186/s12943-020-01150-4 (PMC7045492; doi:10.1186/s12943-020-01150-4)
Supplement: Supplementary file 1 — Additional file 1. Supplementary methods [file 12943_2020_1150_MOESM1_ESM.doc]

# Cell culture and authentication

Cell lines were cultured in RPMI 1640 medium or DMEM containing 10% FBS and 100 U/mL penicillin/streptomycin (Invitrogen, CA, USA) at 37 °C in a humidified incubator with 5% CO_2_. These cell lines were tested and authenticated by Beijing JianLian Genes Technology Co., LTD before they were used in this study. STR patterns were analyzed using Goldeneye^TM^20A STR Identifiler PCR Amplification Kit. Gene Mapper v3.2 software (ABI) was used to match the STR pattern with the online databases of National Platform of Experimental Cell Resources for Sci-Tech for BGC823, MGC803, and SGC7901 cells cell and the American Type Culture Collection (ATCC) for other cells.

**Western blotting**

The cells were collected and lysed to obtain protein lysate. The resulting proteins were then electrophoresed through a 10% SDS-PAGE gel and transferred onto a PVDF membrane. After blocking with 5% fat-free milk overnight at 4 °C, the membrane was incubated with the primary antibody (anti-P16, Abcam, ab81278; anti-P15, Abcam, ab53034; anti-P14, Abcam, ab3642; anti-AUF1, Abcam, ab61193, UK; anti-GAPDH, Protein Tech, 60004-1, China) for 1 hr at room temperature. The membrane was then washed 3 times with PBST (PBS with 0.1% Tween 20). After washing, the membrane was incubated with the corresponding horseradish peroxidase-conjugated goat anti-goat or anti-mouse IgG at room temperature for 1 hr. The signals were visualized using the Immobilon Western Chemiluminescent HRP Substrate Kit (WBKLS0500, Millipore, Billerica, USA).

**RNA extraction, RT-PCR, and quantitative RT-PCR (qRT-PCR) assays**

Total RNA was isolated using the Ultrapure RNA kit (Beijing Com Win Biotech Co., Ltd, China). The cDNAs were synthesized using the first-cDNA synthesis kit (Transgen Co, Beijing, China) according to the manufacturer's instructions. The total RNA was predigested with RNase *R* (RNR07250, Epicentre, USA) before cDNA synthesis for excluding the linear RNAs. The qRT-PCR assay was performed using SYBR Green PCR master mix reagents (FastStart Universal SYBR Green Master, Roche, Mannheim, Germany). The expression levels of *P14AS* and the protein-coding genes were normalized to those of *GAPDH* (for cultured cells) or *Alu* (for tissues). The relative mRNA level was calculated using the typical ΔΔCt method. When *P14AS* and *ANRIL* RNA signals were detected in a sample, the sample was defined as *P14AS* and *ANRIL* coexpression-positive.

# RNA sequencing

The AUF1 gene was knocked down in stably *P14AS*-overexpressed HCT116 cells for 72 hrs. Total RNAs were extracted with the Trizol reagent, amplified, and transcribed into fluorescent cRNA. The purified library products were diluted to 10 pM. RNA-Seq was performed using an Illumina HiSeq 2500 (Illumina, Santiago, CA, USA) at RiboBio Co., Ltd. (Guangzhou, China). Two repeat samples were sequenced for each treatment group. When the average fold change for a gene was >2.0 and the read values between two groups were not overlapped, it was defined as differentially expressed gene. The data sets were deposited in Gene Expression Omnibus database with the accession number GSE127905. Function annotation for differentially expressed genes was performed using the David 6.8 online tools at the website (https://david.ncifcrf.gov/tools.jsp) (11).

**RNA-FISH assay**

Fluorescence-conjugated *P14AS* probes were generated according to protocols from RiboBio Co., Ltd (Guangzhou, China). Cells pretreated with 4% paraformaldehyde were hybridized with RNA probe sets labeled with Cy3, and then stained with DAPI using the RiboTM Fluorescent In Situ Hybridization kit (C10910, Guangzhou RiboBio Co., Ltd., China) according to the manufacturer’s instructions. Images were obtained with a confocal microscope (Olympus, Japan). In addition, *18S* rRNA and *U6* RNA were used as cytoplasm and nucleus RNA controls, respectively.

**RNA pull-down assay**

Biotin-labeled targeted *P14AS* probe #1-#6, and control probe #1-#2 (*Escherichia coli* strain genome) (Table 1) were synthesized using PierceTM RNA 3′ End Desthiobiotinylation Kit (20163, Thermo Scientific, Rockford, IL, USA) by the Beijing Genomics Institute *in vitro*, followed by incubation with separated lysates from HEK293T cells. Activated Streptavidin-Dynabeads (11205D, Thermo Fisher Scientific, Waltham, MA , USA) were coated with 10 μL per sample yeast tRNA (10 mg/mL stock; Ambion, Austin, USA) and 10 μL BSA (10 ng/mL stock), and were incubated in lysis buffer (480 μL) with rotation at 4 °C for 0.5 hr. The beads were then washed, and the sample lysates (600 μL) were mixed with precoated beads (50 μL per sample) and incubated at 4 °C for 4 hrs on a rotator. The beads were then pelleted down the next day to remove unbound materials at 4 °C for 2 min at 1500 rpm and washed six times with 500 μL of ice-cold lysis buffer. Pull-down components were separated by SDS–PAGE followed by immunoblotting with anti-AUF1 antibody or silver staining (24600, Pierce Silver Stain for Mass Spectrometry, Thermo Scientific, Rockford, IL, USA). Differential bands enriched by *P14AS* were analyzed by mass spectrometry. Truncated fragments of *P14AS* were transcribed *in vitro* (P1460, Promega, Madison, WI, USA) and labeled with PierceTM RNA 3′ End Desthiobiotinylation Kit (20163, Thermo Scientific, Rockford, IL, USA) followed by RNA pull-down and immunoblotting.

**Cell proliferation and migration assays using IncuCyte**

HCT116, SW480, and MGC803 cells were seeded into 96-well plates (2,000 cells/well, 5 wells/group) and cultured for at least 96 hrs to determine the proliferation curves. The cells were photographed every 6 hrs in the long-term dynamic observation platform (IncuCyte, Essen, MI, USA). The cell confluence was analyzed using IncuCyte ZOOM software (Essen, Ann Arbor, MI, USA). For continuous observation of cell migration, the cells were seeded into 96-well plates at a density of 10,000 cells/well and cultured for 24 hrs. After a wound was scratched, the cells were washed three times with PBS. The cells were regularly cultured and photographed every 6 hrs for at least 96 hrs. The relative wound width was calculated using the same software.

**Cell proliferation CCK-8 assay**

100μl cell suspension (10000 cells/ml) was dispensed in each well on two 96-well plates and 6 wells for each treatment. The cell proliferation was analyzed by cell counting kit-8 (CCK-8, Dojindo, China). Briefly, add 10μl CCK-8 solution to each well of the plate and incubate the plate for 1 hour in the 37 °C incubator, and then measure the absorbance at 450nm using BioRad microplate reader. The cells were measured daily for 5 continuous days. The average absorbance was calculated.

**Downregulation of genes by siRNA**

All siRNAs used (Table 1) were chemically synthesized by GenePharma Co., Ltd. (Shanghai, China). When HCT116 cells reached a confluence of approximately 60-80%, the cells were transfected with the siRNAs using X-tremeGENE^TM^ siRNA Transfection Reagent (Roche, Mannheim, Germany) according to the manufacturer’s manual. Successful knockdown of AUF1 and *P14AS* expression was confirmed by Western blotting and qRT-PCR, respectively. LV10-NC and LV-shAUF1 lentiviral vectors were used for stable AUF1 knockdown experiments according to the manufacturer’s manual of the lentiviral packaging kit (BG20401S, Beijing Syngentech Co., Ltd., China). Then, puromycin (Sigma, USA) was added into the medium (final concentration, 1 µg/mL) to remove non-transfected cells. The pooled cells treated with puromycin for four weeks were considered to be stably transfected cells.

**Electrophoretic mobility shift assay (EMSA)**

For RNA-EMSAs, 80-nt biotin-labeled *P14AS* RNA probe and unlableled control RNA probes were synthesized by Thermo Fisher Scientific (Waltham, MA, USA; Table S1). LightShift Chemiluminescent RNA-EMSA Kit (Thermo Scientific, Rockford, IL, USA) was used for the shift assay according to manufacturer’s instructions. The 10× unlabeled control probe was used for competitive reaction.

**Induction of methylation of *P16* CpG islands**

Methylation of *P16* CpG islands in BGC823 cells was induced using an engineered *P16-*specific zinc-protein-based DNA methyltransferease (P16-Dnmt) as we previously reported [27].
