# Supplementary material for: Characterization of novel LncRNA P14AS as a protector of ANRIL through AUF1 binding in human cells
Source: Mol Cancer. 2020 Feb 27;19:42. doi: 10.1186/s12943-020-01150-4 (PMC7045492; doi:10.1186/s12943-020-01150-4)
Supplement: Supplementary file 2 — Additional file 2 Figure S1. Characterization of P14AS in the CDKN2A/B locus. (A) Sashimi view for transcripts detected by CDKN2A-specific probe-captured RNA (RNACap)-Seq in HEK293T cells containing two wild-type CDKN2A/B alleles and in MCF7 cells with the homogenous P16 deletion. (B) Graphic view of the protein-coding potential for P14AS, CDKN2A/P14, and ANRIL genes (adapted from the UCSC website). [file 12943_2020_1150_MOESM2_ESM.docx]

**
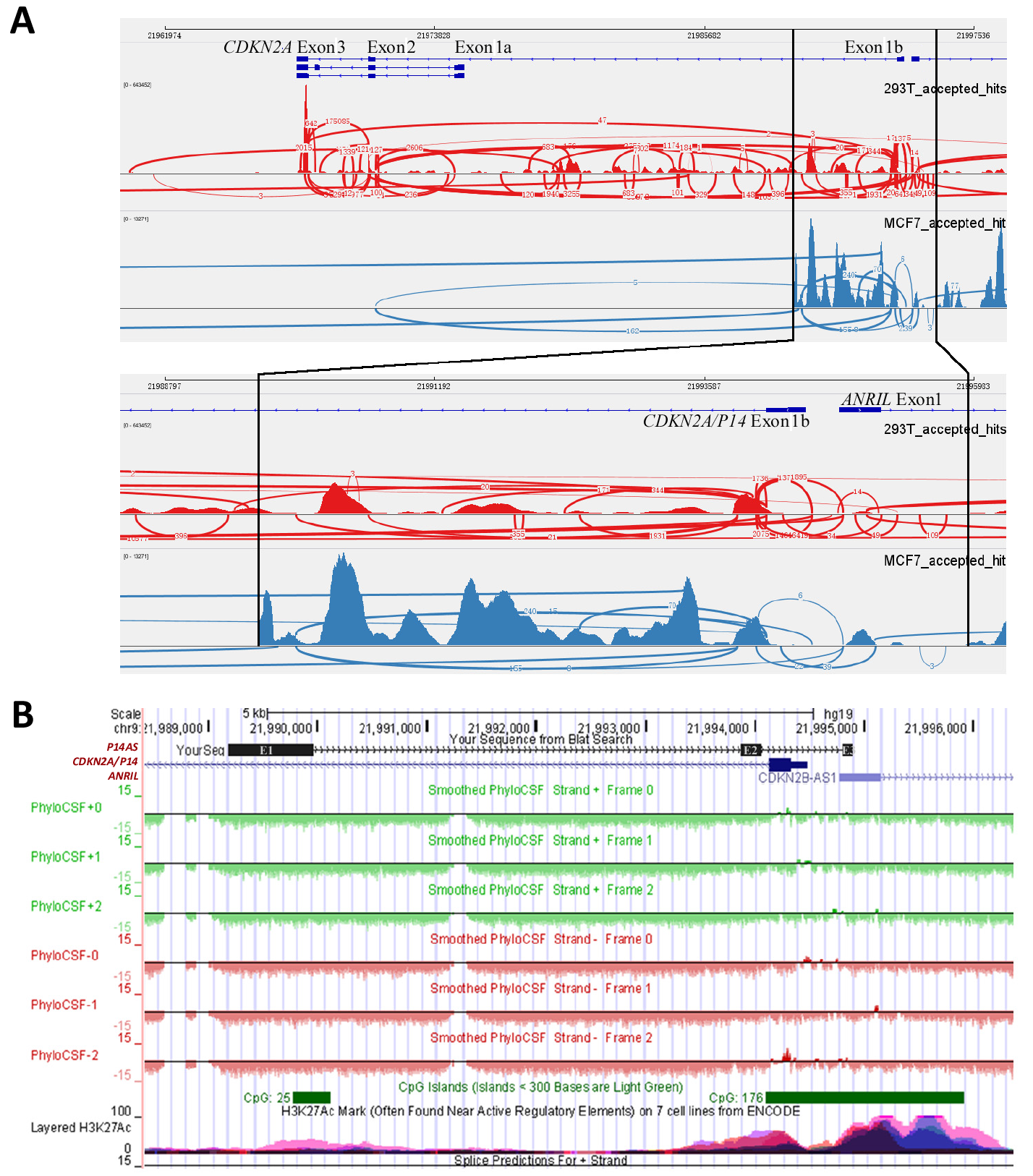
**

**Additional file 2: Fig. S1.** Characterization of *P14AS* in the *CDKN2A/B* locus. (A) Sashimi view for transcripts detected by CDKN2A-specific probe-captured RNA (RNACap)-Seq in HEK293T cells containing two wild-type CDKN2A/B alleles and in MCF7 cells with the homogenous *P16* deletion. (B) Graphic view of the protein-coding potential for *P14AS*, *CDKN2A/P14*, and *ANRIL* genes (adapted from the UCSC website).
