# Supplementary material for: Characterization of novel LncRNA P14AS as a protector of ANRIL through AUF1 binding in human cells
Source: Mol Cancer. 2020 Feb 27;19:42. doi: 10.1186/s12943-020-01150-4 (PMC7045492; doi:10.1186/s12943-020-01150-4)
Supplement: Supplementary file 3 — Additional file 3 Table S1. List of proteins interacted with P14AS characterized in the HEK293T cells in RNA pull-down mass spectrum analysis [file 12943_2020_1150_MOESM3_ESM.docx]

**Additional file 3: Table S1**. List of proteins interacted with P14AS characterized in the HEK293T cells in RNA pull-down mass spectrum analysis

| **Description** | **Mass** | **Score** | **Matches** | **Sequences** |
| --- | --- | --- | --- | --- |
| Heterogeneous nuclear ribonucleoprotein D0 OS=Homo sapiens OX=9606 GN=HNRNPD PE=1 SV=1 | 38581 | 4868 | 293(203) | 32(27) |
| Keratin, type II cytoskeletal 1 OS=Homo sapiens OX=9606 GN=KRT1 PE=1 SV=6 | 66170 | 3897 | 173(124) | 44(40) |
| Keratin, type I cytoskeletal 10 OS=Homo sapiens OX=9606 GN=KRT10 PE=1 SV=6 | 59020 | 2855 | 129(96) | 31(31) |
| Keratin, type II cytoskeletal 2 epidermal OS=Homo sapiens OX=9606 GN=KRT2 PE=1 SV=2 | 65678 | 1908 | 94(68) | 37(29) |
| Actin, cytoplasmic 1 OS=Homo sapiens OX=9606 GN=ACTB PE=1 SV=1 | 42052 | 1798 | 110(74) | 20(18) |
| Keratin, type I cytoskeletal 9 OS=Homo sapiens OX=9606 GN=KRT9 PE=1 SV=3 | 62255 | 1645 | 96(61) | 27(22) |
| Heterogeneous nuclear ribonucleoprotein A/B OS=Homo sapiens OX=9606 GN=HNRNPAB PE=1 SV=2 | 36316 | 1589 | 97(61) | 13(10) |
| Keratin, type I cytoskeletal 14 OS=Homo sapiens OX=9606 GN=KRT14 PE=1 SV=4 | 51872 | 1552 | 83(58) | 30(24) |
| Keratin, type I cytoskeletal 16 OS=Homo sapiens OX=9606 GN=KRT16 PE=1 SV=4 | 51578 | 1124 | 55(40) | 21(15) |
| Actin, alpha cardiac muscle 1 OS=Homo sapiens OX=9606 GN=ACTC1 PE=1 SV=1 | 42334 | 1050 | 73(45) | 12(12) |
| Heterogeneous nuclear ribonucleoprotein K OS=Homo sapiens OX=9606 GN=HNRNPK PE=1 SV=1 | 51230 | 947 | 58(41) | 16(15) |
| Glyceraldehyde-3-phosphate dehydrogenase OS=Homo sapiens OX=9606 GN=GAPDH PE=1 SV=3 | 36201 | 870 | 62(30) | 20(13) |
| Interleukin enhancer-binding factor 2 OS=Homo sapiens OX=9606 GN=ILF2 PE=1 SV=2 | 43263 | 869 | 44(31) | 14(11) |
| Heterogeneous nuclear ribonucleoproteins C1/C2 OS=Homo sapiens OX=9606 GN=HNRNPC PE=1 SV=4 | 33707 | 824 | 46(30) | 12(10) |
| Keratin, type I cytoskeletal 17 OS=Homo sapiens OX=9606 GN=KRT17 PE=1 SV=2 | 48361 | 824 | 49(36) | 20(16) |
| Poly(rC)-binding protein 2 OS=Homo sapiens OX=9606 GN=PCBP2 PE=1 SV=1 | 38955 | 803 | 39(26) | 12(9) |
| Keratin, type II cytoskeletal 5 OS=Homo sapiens OX=9606 GN=KRT5 PE=1 SV=3 | 62568 | 791 | 56(35) | 25(18) |
| Poly(rC)-binding protein 1 OS=Homo sapiens OX=9606 GN=PCBP1 PE=1 SV=2 | 37987 | 780 | 47(30) | 12(10) |
| Keratin, type I cytoskeletal 19 OS=Homo sapiens OX=9606 GN=KRT19 PE=1 SV=4 | 44079 | 694 | 42(29) | 19(12) |
| X-ray repair cross-complementing protein 6 OS=Homo sapiens OX=9606 GN=XRCC6 PE=1 SV=2 | 70084 | 687 | 54(31) | 21(16) |
| Heterogeneous nuclear ribonucleoprotein D-like OS=Homo sapiens OX=9606 GN=HNRNPDL PE=1 SV=3 | 46580 | 675 | 42(30) | 7(6) |
| Proliferation-associated protein 2G4 OS=Homo sapiens OX=9606 GN=PA2G4 PE=1 SV=3 | 44101 | 627 | 65(33) | 20(15) |
| Elongation factor Tu, mitochondrial OS=Homo sapiens OX=9606 GN=TUFM PE=1 SV=2 | 49852 | 605 | 59(31) | 23(16) |
