# Supplementary material for: Characterization of novel LncRNA P14AS as a protector of ANRIL through AUF1 binding in human cells
Source: Mol Cancer. 2020 Feb 27;19:42. doi: 10.1186/s12943-020-01150-4 (PMC7045492; doi:10.1186/s12943-020-01150-4)
Supplement: Supplementary file 4 — Additional file 4 Figure S2.P14AS affects the expression level of the neighboring genes at 9p21.3. qRT-PCR data are normalized to GAPDH mRNA levels and shown as the means ± SD. (a) The expression levels of P14, P16, and P15 genes in the P14AS vector stably transfected colon cancer cells (HCT116 and SW480), and gastric cancer cells (MGC803) were analyzed by qRT-PCR. (b) Detection of P16/P15/P14 proteins in MGC803 cells in Western blot analyses. (c) A fragment deletion in P14AS exon 1 was detected by PCR (top chart) in HEK293T or HCT116 P14AS-KO clones (KO). The expression changes of P14, P16, and P15 genes in HCT116 and HEK293T cells whose ARE-containing elements in P14AS exon-1 were homogenously deleted in the qRT-PCR analysis (bottom chart). Pooled P14AS ARE-KO-negative subclones were used as a wild-type (WT) control. (d) Detection of the P16, P15, and P14 proteins in HEK293T cells in Western blot analyses. [file 12943_2020_1150_MOESM4_ESM.docx]

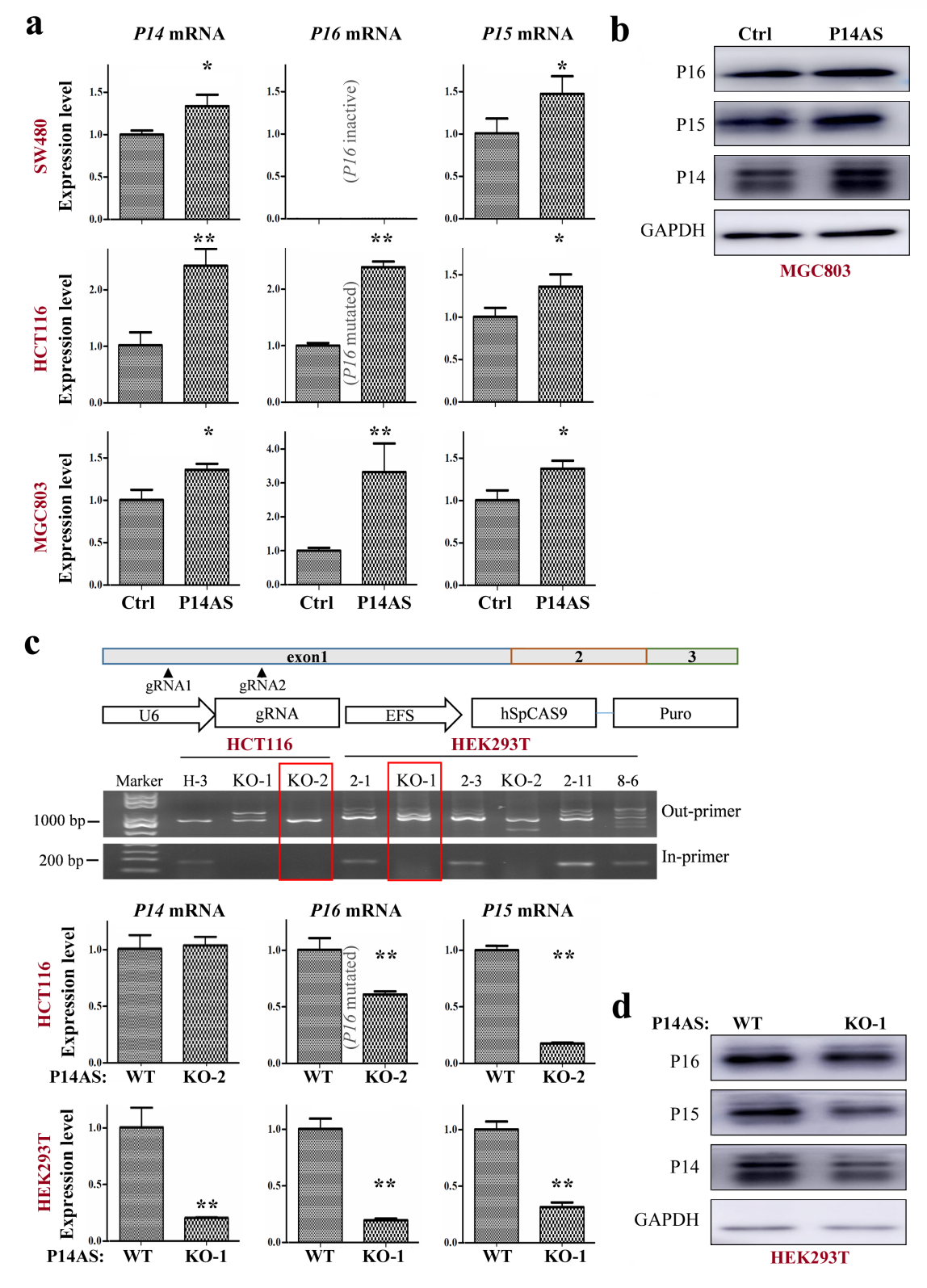


**Additional file 4: Fig. S2.** *P14AS* affects the expression level of the neighboring genes at 9p21.3. qRT-PCR data are normalized to *GAPDH* mRNA levels and shown as the means ± SD. (**a**) The expression levels of *P14*, *P16*, and *P15* genes in the *P14AS* vector stably transfected colon cancer cells (HCT116 and SW480), and gastric cancer cells (MGC803) were analyzed by qRT-PCR. (**b**) Detection of P16/P15/P14 proteins in MGC803 cells in Western blot analyses. (**c**) A fragment deletion in *P14AS* exon 1 was detected by PCR (top chart) in HEK293T or HCT116 *P14AS*-KO clones (KO). The expression changes of *P14*, *P16*, and *P15* genes in HCT116 and HEK293T cells whose ARE-containing elements in *P14AS* exon-1 were homogenously deleted in the qRT-PCR analysis (bottom chart). Pooled *P14AS* ARE-KO-negative subclones were used as a wild-type (WT) control. (**d**) Detection of the P16, P15, and P14 proteins in HEK293T cells in Western blot analyses.
