# Supplementary material for: Characterization of novel LncRNA P14AS as a protector of ANRIL through AUF1 binding in human cells
Source: Mol Cancer. 2020 Feb 27;19:42. doi: 10.1186/s12943-020-01150-4 (PMC7045492; doi:10.1186/s12943-020-01150-4)
Supplement: Supplementary file 5 — Additional file 5 Figure S3. P14AS expression decreased P16 mRNA-AUF1 binding. (a) AUF1 directly bound to ANRIL and P16 mRNA in HCT116 cells in the AUF1-RIP-PCR. (b) P14AS overexpression decreased ANRIL and P16 mRNA-AUF1 interaction in by the AUF1-RIP-qPCR. (c) An illustration of how the competitive AUF1-P14AS binding protects ANRIL and P16 mRNA from the decay. AUF1 complexes were drawn as dimers based on the reports that AUF1 isoforms (p37, p40, p42, and p45) could form functional dimers [25, 26]. [file 12943_2020_1150_MOESM5_ESM.docx]

**
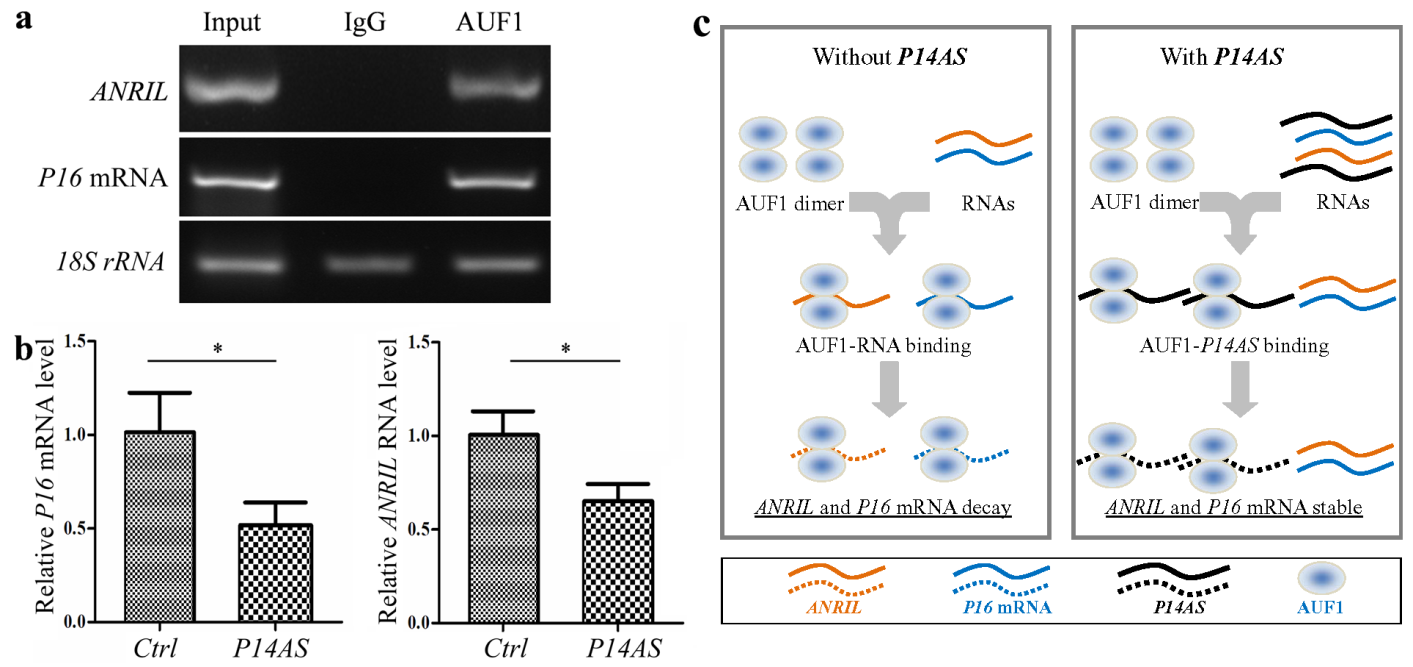
**

**Additional file 5: Fig. S3**. *P14AS* expression decreased *P16* mRNA-AUF1 binding. (**a**) AUF1 directly bound to *ANRIL* and *P16* mRNA in HCT116 cells in the AUF1-RIP-PCR. (**b**) *P14AS* overexpression decreased *ANRIL* and *P16* mRNA-AUF1 interaction in by the AUF1-RIP-qPCR. (**c**) An illustration of how the competitive AUF1-*P14AS* binding protects *ANRIL* and *P16* mRNA from the decay. AUF1 complexes were drawn as dimers based on the reports that AUF1 isoforms (p37, p40, p42, and p45) could form functional dimers (25, 26).
