# Supplementary material for: Characterization of novel LncRNA P14AS as a protector of ANRIL through AUF1 binding in human cells
Source: Mol Cancer. 2020 Feb 27;19:42. doi: 10.1186/s12943-020-01150-4 (PMC7045492; doi:10.1186/s12943-020-01150-4)
Supplement: Supplementary file 6 — Additional file 6 Figure S4. Genome-wide analyses of transcriptome by RNA sequencing for HCT116 cells with and without P14AS overexpression and/or AUF1 downregulation. The HCT116 cells with stable P14AS overexpression were transfected with AUF1 siRNAs (siAUF1) for 72 h, and then harvested for RNA sequencing. The number of genes with > 2 fold changes (UP, upregulated; Down, downregulated) for different types of RNAs were labeled. Western blot analysis for the determination of AUF1 downregulation by siRNAs was inserted into the top chart. Two samples were sequenced for each group. [file 12943_2020_1150_MOESM6_ESM.docx]

**
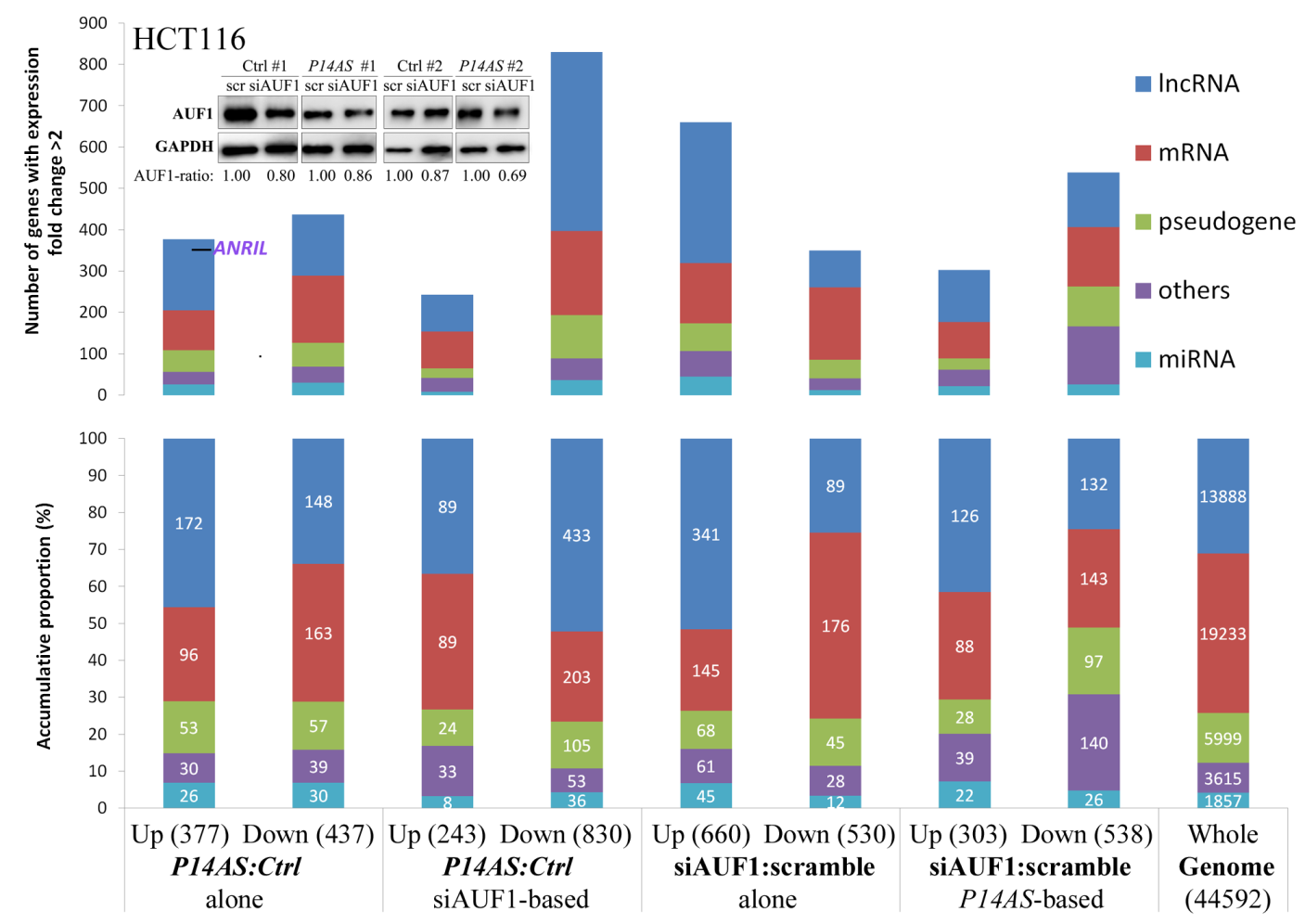
**

**Additional file 6: Fig. S4.** Genome-wide analyses of transcriptome by RNA sequencing for HCT116 cells with and without *P14AS* overexpression and/or AUF1 downregulation. The HCT116 cells with stable *P14AS* overexpression were transfected with *AUF1* siRNAs (siAUF1) for 72 hrs, and then harvested for RNA sequencing. The number of genes with >2 fold changes (UP, upregulated; Down, downregulated) for different types of RNAs were labeled. Western blot analysis for the determination of *AUF1* downregulation by siRNAs was inserted into the top chart. Two samples were sequenced for each group.
