# Supplementary material for: Characterization of novel LncRNA P14AS as a protector of ANRIL through AUF1 binding in human cells
Source: Mol Cancer. 2020 Feb 27;19:42. doi: 10.1186/s12943-020-01150-4 (PMC7045492; doi:10.1186/s12943-020-01150-4)
Supplement: Supplementary file 7 — Additional file 7 Table S2. Function annotations for P14AS-upregulated genes (n = 241) with fold change > 2 in HCT116 cells with the David 6.8: Functional Annotation Tools at the website http://david.ncifcrf.gov/tools.jsp [13] [file 12943_2020_1150_MOESM7_ESM.docx]

**Additional file 7: Table S2**. Function annotations for *P14AS*-upregulated genes (n=241) with fold change >2 in HCT116 cells with the David 6.8: Functional Annotation Tools at the website http://david.ncifcrf.gov/tools.jsp [13]

| **Category** | **Term** | **Count** | **%** | **Genes** | **List Total** | **Pop Hits** | **Pop Total** | **Fold Enrichment** | **FDR** |
| --- | --- | --- | --- | --- | --- | --- | --- | --- | --- |
| UP_KEYWORDS | Glycoprotein | 47 | 19.5 | *CSF2, LY6G6F, CGB3, CGB2, POSTN, GREM1, MANSC1, TMEM178B, SEMA5A, PCDHGB1, TNFRSF11B, DPCR1, LRRTM4, PRRT2, TPO, PLA1A, ANO2, PCDHA11, LOX, ANGPTL3, PRIMA1, ROS1, IL13RA2, PCDHGA12, CRYAB, ACKR2, ACPT, MMP13, MXRA5, THY1, OR51M1, CHRM5, PTGDS, LCTL, ITGA8, LRRC3C, DSG1, COL1A2, ACE2, FBLN7, OR7D2, PLA2G3, TMEM119, SSC5D, NTM, IGFBP4, CDH11* | 101 | 4551 | 20581 | 2.10 | 1.19E-04 |
| UP_SEQ _FEATURE | signal peptide | 39 | 16.2 | *CSF2, LY6G6F, CGB3, CGB2, POSTN, GREM1, MANSC1, SEMA5A, PCDHGB1, TNFRSF11B, DPCR1, LRRTM4, TPO, PLA1A, PTN, PCDHA11, LOX, ANGPTL3, PRIMA1, COL8A1, ROS1, IL13RA2, PCDHGA12, ACPT, MMP13, MXRA5, THY1, PTGDS, LCTL, ITGA8, DSG1, COL1A2, ACE2, FBLN7, TMEM119, PLA2G3, NTM, IGFBP4, CDH11* | 98 | 3346 | 20063 | 2.39 | 1.41E-04 |
| UP_KEYWORDS | Signal | 43 | 17.8 | *LY6G6E, CSF2, LY6G6F, CGB3, CGB2, POSTN, GREM1, MANSC1, TMEM178B, SEMA5A, PCDHGB1, TNFRSF11B, DPCR1, LRRTM4, TPO, PLA1A, PTN, PCDHA11, LOX, ANGPTL3, PRIMA1, COL8A1, ROS1, IL13RA2, PCDHGA12, ACPT, MMP13, MXRA5, THY1, PTGDS, LCTL, ITGA8, LRRC3C, DSG1, COL1A2, ACE2, FBLN7, PLA2G3, TMEM119, SSC5D, NTM, IGFBP4, CDH11* | 101 | 4160 | 20581 | 2.11 | 6.78E-04 |
| UP_SEQ _FEATURE | glycosylation site:N-linked (GlcNAc...) | 43 | 17.8 | *CSF2, LY6G6F, CGB3, CGB2, POSTN, GREM1, MANSC1, SEMA5A, PCDHGB1, TNFRSF11B, DPCR1, LRRTM4, PRRT2, TPO, PLA1A, ANO2, PCDHA11, LOX, ANGPTL3, PRIMA1, ROS1, IL13RA2, PCDHGA12, ACKR2, ACPT, MMP13, MXRA5, THY1, OR51M1, CHRM5, PTGDS, LCTL, ITGA8, DSG1, COL1A2, ACE2, FBLN7, OR7D2, SP9, PLA2G3, NTM, IGFBP4, CDH11* | 98 | 4234 | 20063 | 2.08 | 9.64E-04 |
