# Supplementary material for: Characterization of novel LncRNA P14AS as a protector of ANRIL through AUF1 binding in human cells
Source: Mol Cancer. 2020 Feb 27;19:42. doi: 10.1186/s12943-020-01150-4 (PMC7045492; doi:10.1186/s12943-020-01150-4)
Supplement: Supplementary file 8 — Additional file 8 Table S3. Function annotations for P14AS-downregulated genes (n = 299) with fold change > 2 in HCT116 cells with the David 6.8: Functional Annotation Tools at the website http://david.ncifcrf.gov/tools.jsp [13] [file 12943_2020_1150_MOESM8_ESM.docx]

**Additional file 8: Table S3**. Function annotations for *P14AS*-downregulated genes (n=299) with fold change >2 in HCT116 cells with the David 6.8: Functional Annotation Tools at the website http://david.ncifcrf.gov/tools.jsp [13]

| **Category** | **Term** | **Count** | **%** | **Genes** | **List Total** | **Pop Hits** | **Pop Total** | **Fold Enrichment** | **FDR** |
| --- | --- | --- | --- | --- | --- | --- | --- | --- | --- |
| UP_SEQ _FEATURE | signal peptide | 67 | 22.4 | *ASPN, PLXNA4, COL21A1, PCDHA3, MMP8, ADGRF5, TLR2, MMP3, SIRPB1, STRCP1, CFH, ITIH5, KLK14, LAG3, IHH, CD200R1, F11, ICAM1, PCDHB7, PRG4, SPARCL1, MGP, F7, COLEC11, CD163, PRELP, SLIT3, VEGFD, PLXDC2, CPXM1, CNTN4, ADAMTS1, ADAMTS4, SMIM24, WFIKKN1, CCL2, PAMR1, DCN, CCL28, ABI3BP, TAC3, IGSF10, ZPLD1, DCT, NPIPB10P, FCMR, ALB, HLA-DRB5, TNN, PRSS55, LMAN1L, PTPRC, IL2RB, GNRH2, EFEMP1, ITGA4, ECM2, ADIPOQ, THSD7B, BTLA, LYVE1, FBLN2, PECAM1, SFRP4, CP, ADGRL3, ADGRL4* | 173 | 3346 | 20063 | 2.32 | 1.10E-08 |
| UP_KEYWORDS | Signal | 73 | 24.4 | *ASPN, PLXNA4, COL21A1, PCDHA3, MMP8, ADGRF5, TLR2, MMP3, SIRPB1, STRCP1, KCNQ3, CFH, ITIH5, KLK14, LAG3, IHH, CD200R1, F11, ICAM1, IGLV1-51, PCDHB7, PRG4, SPARCL1, MGP, PKDCC, F7, COLEC11, CD163, PRELP, SLIT3, VEGFD, PLXDC2, CPXM1, ERVH48-1, CNTN4, ADAMTS1, ADAMTS4, SMIM24, WFIKKN1, CCL2, PAMR1, DCN, CCL28, ABI3BP, TAC3, IGSF10, ZPLD1, DCT, SLCO1A2, ALB, FCMR, GALNT15, HLA-DRB5, TNN, PRSS55, PTPRC, LMAN1L, IL2RB, GNRH2, EFEMP1, HBA2, ITGA4, ECM2, ADIPOQ, THSD7B, BTLA, LYVE1, FBLN2, PECAM1, SFRP4, CP, ADGRL3, ADGRL4* | 171 | 4160 | 20581 | 2.11 | 4.89E-08 |
| UP_KEYWORDS | Disulfide bond | 63 | 21.1 | *ASPN, PLXNA4, MMP8, ADGRF5, TLR2, MMP3, SIRPB1, CFH, KLK14, LAG3, CD200R1, F11, ICAM1, IGLV1-51, SPARCL1, PRG4, MGP, F7, COLEC11, CD163, SLIT3, PRELP, VEGFD, SSTR3, CD36, KRT14, CPXM1, CNTN4, ADAMTS1, ADAMTS4, WFIKKN1, CCL2, PAMR1, FPR2, DCN, CCL28, CD74, ZPLD1, IGSF10, SLCO1A2, FCMR, ALB, GALNT15, HLA-DRB5, TNN, TXNIP, PRSS55, LMAN1L, IL2RB, HS3ST3A1, EFEMP1, EDA2R, ITGA4, ADIPOQ, THSD7B, BTLA, LYVE1, FBLN2, SFRP4, PECAM1, CP, ADGRL3, ADGRL4* | 171 | 3434 | 20581 | 2.21 | 4.65E-07 |
| UP_KEYWORDS | Secreted | 45 | 15.1 | *ASPN, WFIKKN1, CCL2, COL21A1, PAMR1, MMP8, DCN, MMP3, CCL28, ABI3BP, TAC3, IGSF10, STRCP1, FCMR, ALB, CFH, ITIH5, TNN, KLK14, CD200R1, IHH, F11, IGLV1-51, GNRH2, SPARCL1, PRG4, EFEMP1, MGP, PKDCC, COLEC11, F7, ECM2, ADIPOQ, CD163, SLIT3, PRELP, VEGFD, FBLN2, SFRP4, CPXM1, ERVH48-1, ADAMTS1, CNTN4, CP, ADAMTS4* | 171 | 1965 | 20581 | 2.76 | 7.11E-07 |
| UP_KEYWORDS | Glycoprotein | 73 | 24.4 | *ASPN, PLXNA4, COL21A1, PCDHA3, MMP8, ADGRF5, TLR2, MMP3, SIRPB1, STRCP1, CFH, ITIH5, LAG3, IHH, CD200R1, F11, ICAM1, PCDHB7, PRG4, SPARCL1, PKDCC, F7, CD163, PRELP, SLIT3, VEGFD, SSTR3, CD36, PLXDC2, CPXM1, CNTN4, ADAMTS1, TM4SF1, ADAMTS4, ABCA8, WFIKKN1, CCL2, PAMR1, DCN, FPR2, CCL28, CD74, ABI3BP, IGSF10, ZPLD1, DCT, SLCO1A2, SYN1, ALB, GALNT15, HLA-DRB5, TNN, HBB, PRSS55, PTPRC, LMAN1L, IL2RB, HS3ST3A1, EFEMP1, EDA2R, HBA2, ITGA4, ECM2, ADIPOQ, THSD7B, BTLA, LYVE1, FBLN2, PECAM1, SFRP4, CP, ADGRL3, ADGRL4* | 171 | 4551 | 20581 | 1.93 | 3.31E-06 |
| UP_SEQ _FEATURE | disulfide bond | 56 | 18.7 | *ASPN, PLXNA4, MMP8, TLR2, MMP3, SIRPB1, CFH, KLK14, LAG3, CD200R1, F11, ICAM1, SPARCL1, PRG4, MGP, F7, COLEC11, CD163, SLIT3, PRELP, VEGFD, SSTR3, CD36, CPXM1, CNTN4, ADAMTS1, ADAMTS4, WFIKKN1, CCL2, PAMR1, FPR2, DCN, CCL28, CD74, IGSF10, FCMR, ALB, GALNT15, HLA-DRB5, TNN, PRSS55, IL2RB, HS3ST3A1, EFEMP1, EDA2R, ITGA4, ADIPOQ, THSD7B, BTLA, LYVE1, FBLN2, SFRP4, PECAM1, CP, ADGRL3, ADGRL4* | 173 | 2917 | 20063 | 2.23 | 7.65E-06 |
| GOTERM_CC _DIRECT | GO:0005576~extracellular region | 38 | 12.7 | *WFIKKN1, CCL2, COL21A1, PAMR1, MMP8, DCN, MMP3, CCL28, TAC3, IGSF10, STRCP1, FCMR, ALB, CFH, ITIH5, HIST1H4I, HBB, CD200R1, F11, IGLV1-51, GNRH2, PRG4, EFEMP1, PKDCC, HBA2, COLEC11, F7, ADIPOQ, CD163, SLIT3, PRELP, VEGFD, S100B, FBLN2, SFRP4, CNTN4, CP, ADAMTS4* | 158 | 1610 | 18224 | 2.72 | 2.95E-05 |
| UP_SEQ _FEATURE | glycosylation site:N-linked (GlcNAc...) | 68 | 22.7 | *ASPN, PLXNA4, COL21A1, PCDHA3, MMP8, ADGRF5, TLR2, MMP3, SIRPB1, STRCP1, CFH, ITIH5, LAG3, IHH, CD200R1, F11, ICAM1, PCDHB7, SPARCL1, PRG4, F7, CD163, PRELP, SLIT3, VEGFD, SSTR3, CD36, PLXDC2, CPXM1, CNTN4, ADAMTS1, TM4SF1, ADAMTS4, ABCA8, WFIKKN1, CCL2, PAMR1, DCN, FPR2, CCL28, CD74, ABI3BP, IGSF10, ZPLD1, DCT, SLCO1A2, NPIPB10P, GALNT15, HLA-DRB5, TNN, PRSS55, LMAN1L, PTPRC, IL2RB, HS3ST3A1, EFEMP1, EDA2R, ITGA4, ECM2, THSD7B, BTLA, LYVE1, FBLN2, PECAM1, SFRP4, CP, ADGRL3, ADGRL4* | 173 | 4234 | 20063 | 1.86 | 1.03E-04 |
| UP_SEQ_FEATURE | repeat:LRR 12 | 10 | 3.3 | *ASPN, IGSF10, NLRC4, NLRC3, TLR2, DCN, ECM2, LRRK2, PRELP, SLIT3* | 173 | 90 | 20063 | 12.89 | 1.23E-04 |
| UP_SEQ_FEATURE | repeat:LRR 11 | 10 | 3.3 | *ASPN, IGSF10, NLRC4, NLRC3, TLR2, DCN, ECM2, LRRK2, PRELP, SLIT3* | 173 | 101 | 20063 | 11.48 | 3.35E-04 |
| GOTERM_CC_DIRECT | GO:0005615~extracellular space | 32 | 10.7 | *CCL2, MMP8, DCN, MMP3, CCL28, ABI3BP, TAC3, ALB, CFH, HLA-DRB5, KLK14, IHH, F11, ICAM1, SPARCL1, EFEMP1, F7, ECM2, ADIPOQ, SLIT3, PRELP, VEGFD, CD36, S100B, SFRP4, CPXM1, PECAM1, SERPINB7, ERVH48-1, CP, LRRK2, ADAMTS4* | 158 | 1347 | 18224 | 2.74 | 5.03E-04 |
| GOTERM_CC_DIRECT | GO:0005578~proteinaceous extracellular matrix | 14 | 4.7 | *ASPN, COL21A1, SPARCL1, MMP8, EFEMP1, MGP, MMP3, ECM2, SLIT3, PRELP, FBLN2, TNN, ADAMTS1, ADAMTS4* | 158 | 268 | 18224 | 6.03 | 7.29E-04 |
| UP_SEQ_FEATURE | repeat:LRR 10 | 10 | 3.3 | *ASPN, IGSF10, NLRC4, NLRC3, TLR2, DCN, ECM2, LRRK2, PRELP, SLIT3* | 173 | 116 | 20063 | 10.00 | 0.001095 |
| UP_KEYWORDS | Extracellular matrix | 13 | 4.3 | *ASPN, COL21A1, SPARCL1, MMP8, EFEMP1, DCN, MMP3, ECM2, PRELP, FBLN2, TNN, ADAMTS1, ADAMTS4* | 171 | 258 | 20581 | 6.06 | 0.002132 |
| UP_SEQ_FEATURE | repeat:LRR 9 | 10 | 3.3 | *ASPN, IGSF10, NLRC4, NLRC3, TLR2, DCN, ECM2, LRRK2, PRELP, SLIT3* | 173 | 134 | 20063 | 8.65 | 0.003674 |
| UP_SEQ_FEATURE | repeat:LRR 8 | 10 | 3.3 | *ASPN, IGSF10, NLRC4, NLRC3, TLR2, DCN, ECM2, LRRK2, PRELP, SLIT3* | 173 | 148 | 20063 | 7.84 | 0.008323 |
| KEGG_PATHWAY | hsa05144:Malaria | 7 | 2.3 | *ICAM1, CD36, CCL2, PECAM1, TLR2, HBA2, HBB* | 68 | 49 | 6879 | 14.45 | 0.008014 |
| GOTERM_MF_DIRECT | GO:0005509~calcium ion binding | 20 | 6.7 | *ASPN, PCDHB7, PCDHA3, SPARCL1, PAMR1, EFEMP1, MMP8, MGP, F7, MMP3, CALB1, SLIT3, RAB44, S100B, FBLN2, HPCA, SYT15, ADGRL3, ADGRL4, IHH* | 144 | 717 | 16881 | 3.271 | 0.01326 |
| UP_SEQ_FEATURE | repeat:LRR 7 | 10 | 3.3 | *ASPN, IGSF10, NLRC4, NLRC3, TLR2, DCN, ECM2, LRRK2, PRELP, SLIT3* | 173 | 176 | 20063 | 6.59 | 0.033612 |
| UP_SEQ_FEATURE | repeat:LRR 5 | 11 | 3.7 | *ASPN, IGSF10, NWD2, NLRC4, NLRC3, TLR2, DCN, ECM2, LRRK2, PRELP, SLIT3* | 173 | 231 | 20063 | 5.52 | 0.049255 |
