# Supplementary material for: Characterization of novel LncRNA P14AS as a protector of ANRIL through AUF1 binding in human cells
Source: Mol Cancer. 2020 Feb 27;19:42. doi: 10.1186/s12943-020-01150-4 (PMC7045492; doi:10.1186/s12943-020-01150-4)
Supplement: Supplementary file 9 — Additional file 9 Figure S5. Association analyses between the expression levels of AUF1 and ANRIL, P16, or P15 using the publicly available transcriptome databases for human cancer cell lines in the CCLE project. (A) All 1037 cell lines; (B) 224 cell lines without the CDKN2A allele deletion (relative copy number > 0). [file 12943_2020_1150_MOESM9_ESM.docx]

**
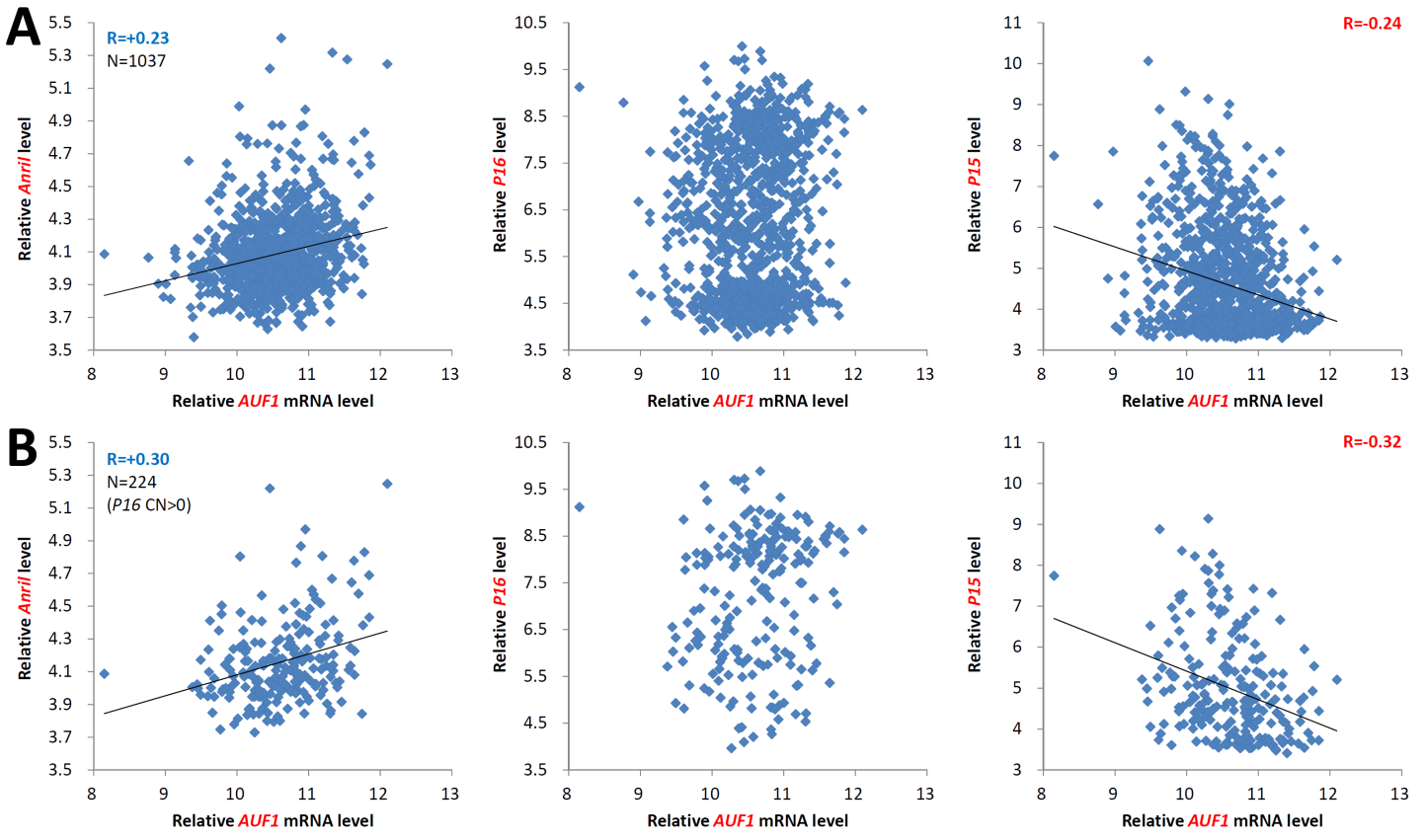
**

**Additional file 9: Fig. S5.** Association analyses between the expression levels of *AUF1* and *ANRIL*, *P16*, or *P15* using the publicly available transcriptome databases for human cancer cell lines in the CCLE project. (**A**) All 1037 cell lines; (**B**) 224 cell lines without the *CDKN2A* allele deletion (relative copy number >0).
