# Supplementary material for: Characterization of novel LncRNA P14AS as a protector of ANRIL through AUF1 binding in human cells
Source: Mol Cancer. 2020 Feb 27;19:42. doi: 10.1186/s12943-020-01150-4 (PMC7045492; doi:10.1186/s12943-020-01150-4)
Supplement: Supplementary file 10 — Additional file 10 Figure S6. Comparison of the levels of P14AS, P16, and P15 expression in colon tissues from cancer and noncancer patients. (a) The expression status of P14AS, P16, and P15 in colon cancer (CC), paired surgical margin (SM), and normal colon biopsy (Normal) tissues from noncancer patients by qRT-PCR. (b) The level of P14AS expression in ANRIL-positive and -negative (by RT-PCR) colon CC and SM tissues. (c and d) Comparison of the expression levels of P16 and P15 mRNA (by qRT-PCR) in P14AS-positive and -negative (by RT-PCR; 1043 bp) colon CC and SM tissues. Error bars, S.E.M. *, P < 0.05; **, P < 0.01; N.S. no significance. [file 12943_2020_1150_MOESM10_ESM.docx]

**
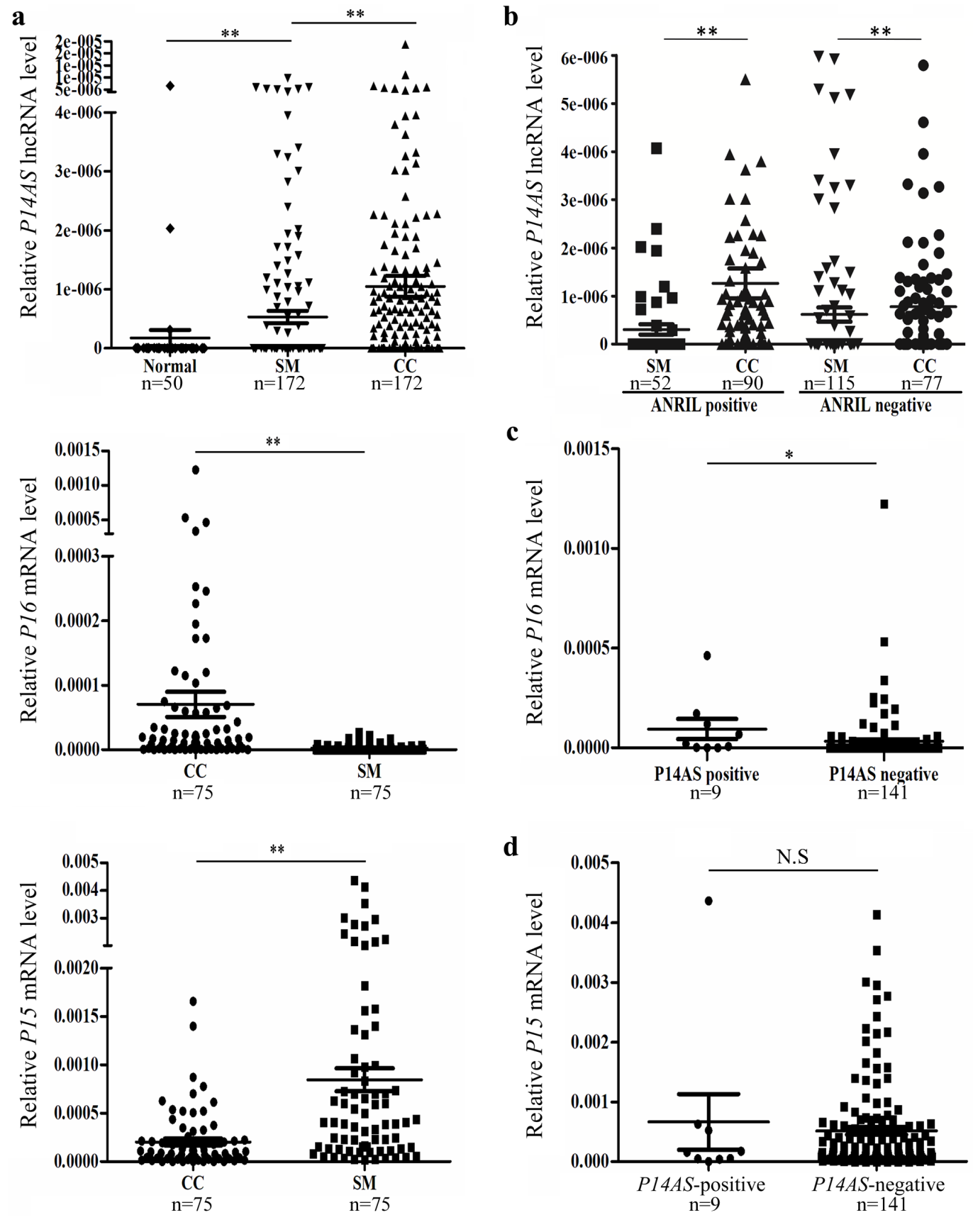
**

**Additional file 10: Fig. S6.** Comparison of the levels of *P14AS*, *P16*, and *P15* expression in colon tissues from cancer and noncancer patients. (**a**) The expression status of *P14AS*, *P16*, and *P15* in colon cancer (CC), paired surgical margin (SM), and normal colon biopsy (Normal) tissues from noncancer patients by qRT-PCR. (**b**) The level of *P14AS* expression in *ANRIL*-positive and -negative (by RT-PCR) colon CC and SM tissues. (**c** and **d**) Comparison of the expression levels of *P16* and *P15* mRNA (by qRT-PCR) in *P14AS*-positive and -negative (by RT-PCR; 1043 bp) colon CC and SM tissues. Error bars, S.E.M. *, *P*<0.05; **, *P*<0.01; N.S. no significance.
