# Supplementary material for: Characterization of novel LncRNA P14AS as a protector of ANRIL through AUF1 binding in human cells
Source: Mol Cancer. 2020 Feb 27;19:42. doi: 10.1186/s12943-020-01150-4 (PMC7045492; doi:10.1186/s12943-020-01150-4)
Supplement: Supplementary file 11 — Additional file 11 Table S4. Comparison of the P14AS expression level (by qRT-PCR) in colon cancer (CC) and surgical margin (SM) tissue samples from patients with different clinicopathological characteristics [file 12943_2020_1150_MOESM11_ESM.docx]

**Additional file 11: Table S4**. Comparison of the *P14AS* expression level (by qRT-PCR) in colon cancer (CC) and surgical margin (SM) tissue samples from patients with different clinicopathological characteristics

|  |  | ***P14AS*-positive rate for CCs (%)** | ***P*-value** | ***P14AS*-positive rate for SMs (%)** | ***P*-value** |
| --- | --- | --- | --- | --- | --- |
| **Age** | <**60** | 32/67 (47.76) | 0.639 | 15/67 (22.39) | 0.830 |
|  | **≥60** | 54/105 (51.43) |  | 25/105 (23.81) |  |
| **Sex** | **Male** | 50/101 (49.51) | 0.949 | 23/101 (22.77) | 0.990 |
|  | **Female** | 35/70 (50.00) |  | 16/70 (22.86) |  |
| **Location** | **Sigmoid** | 44/86 (51.16) | 0.760 | 21/86 (24.42) | 0.718 |
|  | **Others** | 42/86 (48.84) |  | 19/86 (22.09) |  |
| **Differentiation** | **Poor** | 10/18 (55.56) | 0.618 | 2/18 (11.11) | 0.250 |
|  | **Moderate/well** | 76/154 (49.35) |  | 38/154 (24.68) |  |
| **Vascular embolus** | **No** | 68/136 (50.00) | 0.880 | 31/136 (22.79) | 0.994 |
|  | **Yes** | 17/35 (48.57) |  | 8/35 (22.86) |  |
| **pTNM stage** | **I+II** | 37/82 (45.12) | 0.219 | 19/82 (23.17) | 0.945 |
|  | **III+IV** | 48/88 (54.55) |  | 20/88 (22.73) |  |
| **Local invasion** | **T1-2** | 3/12 (25.00) | **0.034** | 4/12 (33.33) | 0.547 |
|  | **T3** | 39/83 (46.99) |  | 17/83 (20.48) |  |
|  | **T4** | 43/75 (57.33) |  | 19/75 (25.33) |  |
| **Lymph metastasis** | **N0** | 38/83 (45.78) | 0.285 | 20/83 (24.10) | 0.801 |
|  | **N1-3** | 48/89 (53.93) |  | 20/89 (22.47) |  |
| **Distant metastasis** | **M0** | 69/141 (48.94) | 0.552 | 37/141 (26.24) | 0.084 |
|  | **M1** | 17/31 (54.84) |  | 3/31 (9.68) |  |
| **(All)** |  | 86/172 (50.00) |  | 40/172 (23.26) | **0.001** |

*: colon cancer *vs*. surgical margin, chi-squared test, odds ratio=3.30, 95% confidence interval: 2.02-5.39
