# Supplementary material for: Characterization of novel LncRNA P14AS as a protector of ANRIL through AUF1 binding in human cells
Source: Mol Cancer. 2020 Feb 27;19:42. doi: 10.1186/s12943-020-01150-4 (PMC7045492; doi:10.1186/s12943-020-01150-4)
Supplement: Supplementary file 12 — Additional file 12 Figure S7. Comparisons of the levels of AUF1 mRNA (by qRT-PCR) with those of P14AS and ANRIL lncRNA in colon cancer tissues (CCs). [file 12943_2020_1150_MOESM12_ESM.docx]

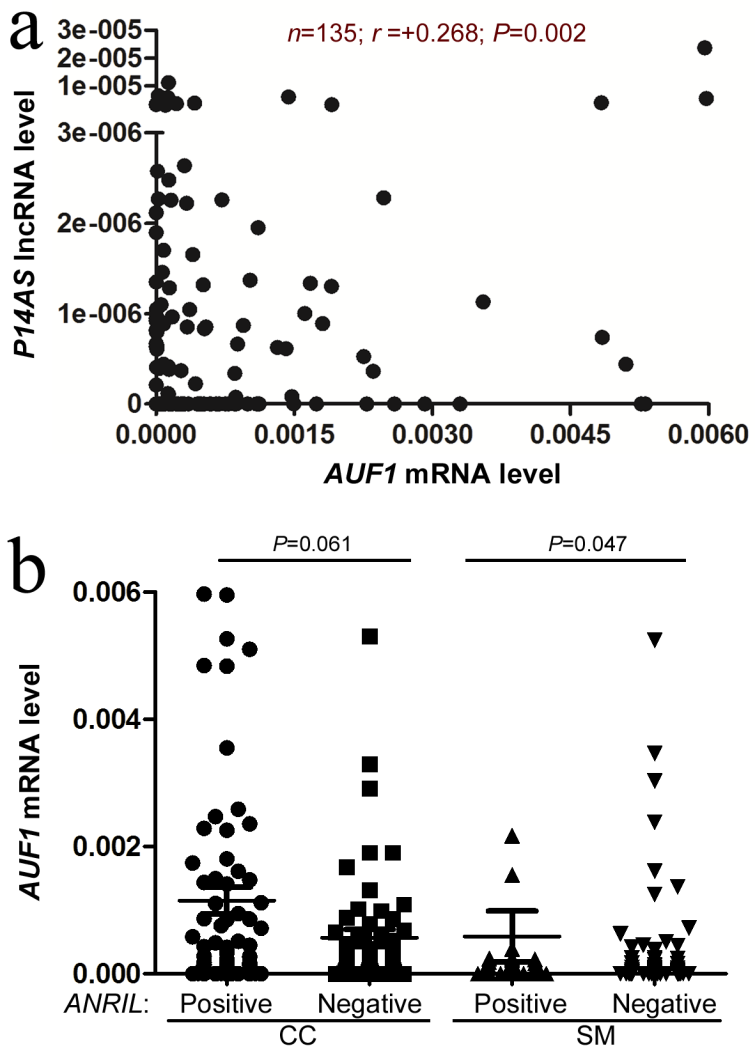


**Additional file 12: Fig. S7.** Comparisons of the levels of *AUF1* mRNA (by qRT-PCR) with those of *P14AS* and *ANRIL* lncRNA in colon cancer tissues (CCs). (**a**) Correlation of the levels of *AUF1* mRNA and P14AS lncRNA in CCs (*N*=140; Pearson_r = +0.27, *P*=0.002) in quantitative qRT-PCR analysis. (**b**) The levels of *AUF1* mRNA in *ANRIL* expression-positive CCs and surgical margin (SM) were higher than those in *ANRIL* expression-negative CCs and SMs. The *P-*values in the Mann–Whitney *U*-test were labeled for these non-normally distributed data.
