# Supplementary material for: Characterization of novel LncRNA P14AS as a protector of ANRIL through AUF1 binding in human cells
Source: Mol Cancer. 2020 Feb 27;19:42. doi: 10.1186/s12943-020-01150-4 (PMC7045492; doi:10.1186/s12943-020-01150-4)
Supplement: Supplementary file 13 — Additional file 13 Table S5. Comparison of the ANRIL expression level (by RT-PCR) in colon cancer (CC) and surgical margin (SM) tissue samples from patients with different clinicopathological characteristics [file 12943_2020_1150_MOESM13_ESM.docx]

**Additional file 13: Table S5**. Comparison of the *ANRIL* expression level (by RT-PCR) in colon cancer (CC) and surgical margin (SM) tissue samples from patients with different clinicopathological characteristics

|  |  | ***ANRIL*-positive rate for CCs (%)** | ***P*-value** | ***ANRIL*-positive rate for SMs (%)** | ***P*-value** |
| --- | --- | --- | --- | --- | --- |
| **Age** | <**60** | 37/65 (56.9) | 0.531 | 21/65 (32.3) | 0.794 |
|  | **≥60** | 53/102 (52.0) |  | 31/102 (30.4) |  |
| **Sex** | **Male** | 46/97 (47.4) | 0.058 | 31/97 (32.0) | 0.835 |
|  | **Female** | 43/69 (62.3) |  | 21/69 (30.4) |  |
| **Location** | **Sigmoid** | 42/83 (50.6) | 0.397 | 22/83 (26.5) | 0.199 |
|  | **Others** | 48/84 (57.1) |  | 30/84 (35.7) |  |
| **Differentiation** | **Poor** | 8/17 (47.1) | 0.551 | 4/17 (23.5) | 0.475 |
|  | **Moderate/well** | 82/150 (54.7) |  | 48/150 (32.0) |  |
| **Vascular embolus** | **No** | 67/131 (51.2) | 0.217 | 43/131 (32.8) | 0.420 |
|  | **Yes** | 22/35 (62.9) |  | 9/35 (25.7) |  |
| **pTNM stage** | **I+II** | 40/80 (50.0) | 0.325 | 26/80 (32.5) | 0.668 |
|  | **III+IV** | 49/85 (57.7) |  | 25/85 (29.4) |  |
| **Local invasion** | **T1-2** | 4/12 (33.3) | 0.136 | 5/12 (41.7) | **0.020** |
|  | **T3** | 48/79 (60.8) |  | 32/79 (40.5) |  |
|  | **T4** | 37/74 (50.0) |  | 15/74 (20.3) |  |
| **Lymph metastasis** | **N0** | 42/81 (51.9) | 0.608 | 25/81 (30.9) | 0.941 |
|  | **N1-3** | 48/86 (55.8) |  | 27/86 (31.4) |  |
| **Distant metastasis** | **M0** | 69/137 (50.4) | **0.051** | 39/137 (28.5) | 0.111 |
|  | **M1** | 21/30 (70.0) |  | 13/30 (43.3) |  |
| **(All)** |  | 90/167 (53.9) |  | 52/167 (31.1) | **0.001*** |

*: colon cancer *vs*. surgical margin, chi-squared test, odds ratio=2.58, 95% confidence interval: 1.61-4.15
