# Supplementary material for: Characterization of novel LncRNA P14AS as a protector of ANRIL through AUF1 binding in human cells
Source: Mol Cancer. 2020 Feb 27;19:42. doi: 10.1186/s12943-020-01150-4 (PMC7045492; doi:10.1186/s12943-020-01150-4)
Supplement: Supplementary file 14 — Additional file 14 Table S6. Comparison of the AUF1 mRNA level (by qRT-PCR) in colon cancer (CC) and surgical margin (SM) tissue samples from patients with different clinicopathological characteristics [file 12943_2020_1150_MOESM14_ESM.docx]

**Additional file 14: Table S6**. Comparison of the *AUF1* mRNA level (by qRT-PCR) in colon cancer (CC) and surgical margin (SM) tissue samples from patients with different clinicopathological characteristics

|  |  | **Case no.** | ***AUF1* mRNA level for CC (*median* [25-75%])** | ***P*-value (U-test)** | ***AUF1* mRNA level for SM (*median* [25-75%])** | ***P*-value (U-test)** |
| --- | --- | --- | --- | --- | --- | --- |
| **Age** | <**60** | 48 | 14.93 [0.74-51.92] | 0.074 | 1.17 [0.05-17.54] | 0.897 |
|  | **≥60** | 87 | 33.94 [2.65-110.87] |  | 1.86 [0.004-22.16] |  |
| **Sex** | **Male** | 77 | 15.70 [1.49-62.57] | 0.179 | 3.20 [0.03-22.27] | 0.717 |
|  | **Female** | 57 | 36.48 [3.32-144.39] |  | 1.17 [0.00-16.51] |  |
| **Location** | **Sigmoid** | 64 | 22.15 [1.44-76.10] | 0.561 | 5.81 [0.18-24.80] | **0.014** |
|  | **Others** | 71 | 17.68 [2.65-110.87] |  | 0.52 [0.00-11.36] |  |
| **Differentiation** | **Poor** | 13 | 8.19 [0.02-32.31] | 0.087 | 0.65 [0.00-4.11] | 0.135 |
|  | **Moderate/well** | 122 | 23.63 [2.24-106.71] |  | 1.94 [0.02-22.21] |  |
| **Vascular embolus** | **No** | 104 | 22.15 [1.97-96.70] | 0.765 | 1.77 [0.01-22.33] | 0.910 |
|  | **Yes** | 30 | 14.94 [1.54-97.63] |  | 3.13 [0.07-16.12] |  |
| **pTNM stage** | **I+II** | 61 | 27.30 [57.21-164.48] | 0.058 | 5.81 [0.17-30.64] | **0.014** |
|  | **III+IV** | 73 | 16.22 [0.95-75.15] |  | 0.53 [0.00-11.69] |  |
| **Local invasion** | **T1-2** | 9 | 7.76 [2.27-118.28] | 0.796 | 17.71 [1.45-13220] | 0.171 |
|  | **T3** | 60 | 19.85 [1.62-105.00] |  | 3.77 [0.08-21.90] |  |
|  | **T4** | 64 | 26.00 [2.78-98.29] |  | 0.73 [0.00-16.37] |  |
| **Lymph metastasis** | **N0** | 62 | 27.02 [5.36-139.20] | 0.100 | 5.57 [0.16-27.72] | **0.021** |
|  | **N1-3** | 73 | 16.23 [0.95-81.91] |  | 0.53 [0.00-11.69] |  |
| **Distant metastasis** | **M0** | 105 | 22.02 [1.77-105.58] | 0.515 | 1.94 [0.02-22.68] | 0.493 |
|  | **M1** | 30 | 24.76 [2.26-70.58] |  | 1.13 [0.01-14.87] |  |
| **(All)** |  | 135 | 22.02 [1.84-99.43] |  | 1.77 [0.02-21.17] | **<0.001*** |

* colon cancer (CC) *vs*. surgical margin (SM)
