# Supplementary material for: Characterization of novel LncRNA P14AS as a protector of ANRIL through AUF1 binding in human cells
Source: Mol Cancer. 2020 Feb 27;19:42. doi: 10.1186/s12943-020-01150-4 (PMC7045492; doi:10.1186/s12943-020-01150-4)
Supplement: Supplementary file 15 — Additional file 15 Table S7. Comparison of P14AS and ANRIL coexpression in colon cancer (CC) and surgical margin (SM) tissue samples from patients with different clinicopathological characteristics [file 12943_2020_1150_MOESM15_ESM.docx]

**Additional file 15: Table S7**. Comparison of *P14AS* and *ANRIL* coexpression in colon cancer (CC) and surgical margin (SM) tissue samples from patients with different clinicopathological characteristics

|  |  |  | ***P14AS*&*ANRIL* co-expression in CC** | | ***P14AS*&*ANRIL* co-expression in SM** | |
| --- | --- | --- | --- | --- | --- | --- |
|  |  | **n** | **Positive case no. (%)** | ***P*-value*** | **Positive case no. (%)** | ***P*-value** |
| **Age** | <**60** | 65 | 21 (32.3) | 0.600 | 5 (7.7) | 0.646 |
|  | **≥60** | 102 | 37 (36.3) |  | 6 (5.9) |  |
| **Sex** | **Male** | 97 | 34 (35.1) | 0.818 | 8 (8.2) | 0.320 |
|  | **Female** | 69 | 23 (33.3) |  | 3 (4.3) |  |
| **Location** | **Sigmoid** | 83 | 29 (34.9) | 0.955 | 6 (7.2) | 0.740 |
|  | **Others** | 84 | 29 (34.5) |  | 5 (6.0) |  |
| **Differentiation** | **Poor** | 17 | 5 (29.4) | 0.627 | 0 (0.0) | 0.248 |
|  | **Mod./well** | 150 | 53 (35.3) |  | 11 (7.3) |  |
| **Vascular embolus** | **No** | 35 | 16 (45.7) | 0.111 | 3 (8.6) | 0.603 |
|  | **Yes** | 131 | 41 (31.3) |  | 8 (6.1) |  |
| **pTNM stage** | **I+II** | 80 | 22 (27.5) | 0.065 | 3 (3.8) | 0.228 |
|  | **III+IV** | 85 | 35 (41.2) |  | 7 (8.2) |  |
| **Local invasion** | **T1-2** | 12 | 2 (16.7) |  | 2 (16.7) |  |
|  | **T3** | 79 | 28 (35.4) | 0.397 | 4 (5.1) | 0.324 |
|  | **T4** | 74 | 27 (36.5) |  | 5 (6.8) |  |
| **Lymph metastasis** | **N0** | 81 | 24 (29.6) | 0.179 | 4 (4.9) | 0.405 |
|  | **N1-3** | 86 | 34 (39.5) |  | 7 (8.1) |  |
| **Distant metastasis** | **M0** | 137 | 42 (30.7) | **0.018** | 9 (6.6) | 0.984 |
|  | **M1** | 30 | 16 (53.3) |  | 2 (6.7) | 0.984 |
| **(All)** |  | 167 | 58 (34.7) |  | 11 (6.6) | **0.001**** |

* Chi-square test; ** CC vs. SM
