# Supplementary material for: Characterization of novel LncRNA P14AS as a protector of ANRIL through AUF1 binding in human cells
Source: Mol Cancer. 2020 Feb 27;19:42. doi: 10.1186/s12943-020-01150-4 (PMC7045492; doi:10.1186/s12943-020-01150-4)
Supplement: Supplementary file 16 — Additional file 16 Figure S8. Graph of the P14AS gene in the CDKN2A/B locus. (A) CpG islands within the CDKN2A/P14 gene. (B) The transcription and active histone modification status in the chromatin upstream of the P14AS gene in 7 cell lines from ENCODE. (C) Transcription factors binding to various fragments around the P14AS gene from ENCODE. The RNA polymerase II (POLR2A) is highlighted in red lines. (D) The conservation status of various fragments among vertebrates (adapted from the UCSC website). [file 12943_2020_1150_MOESM16_ESM.docx]

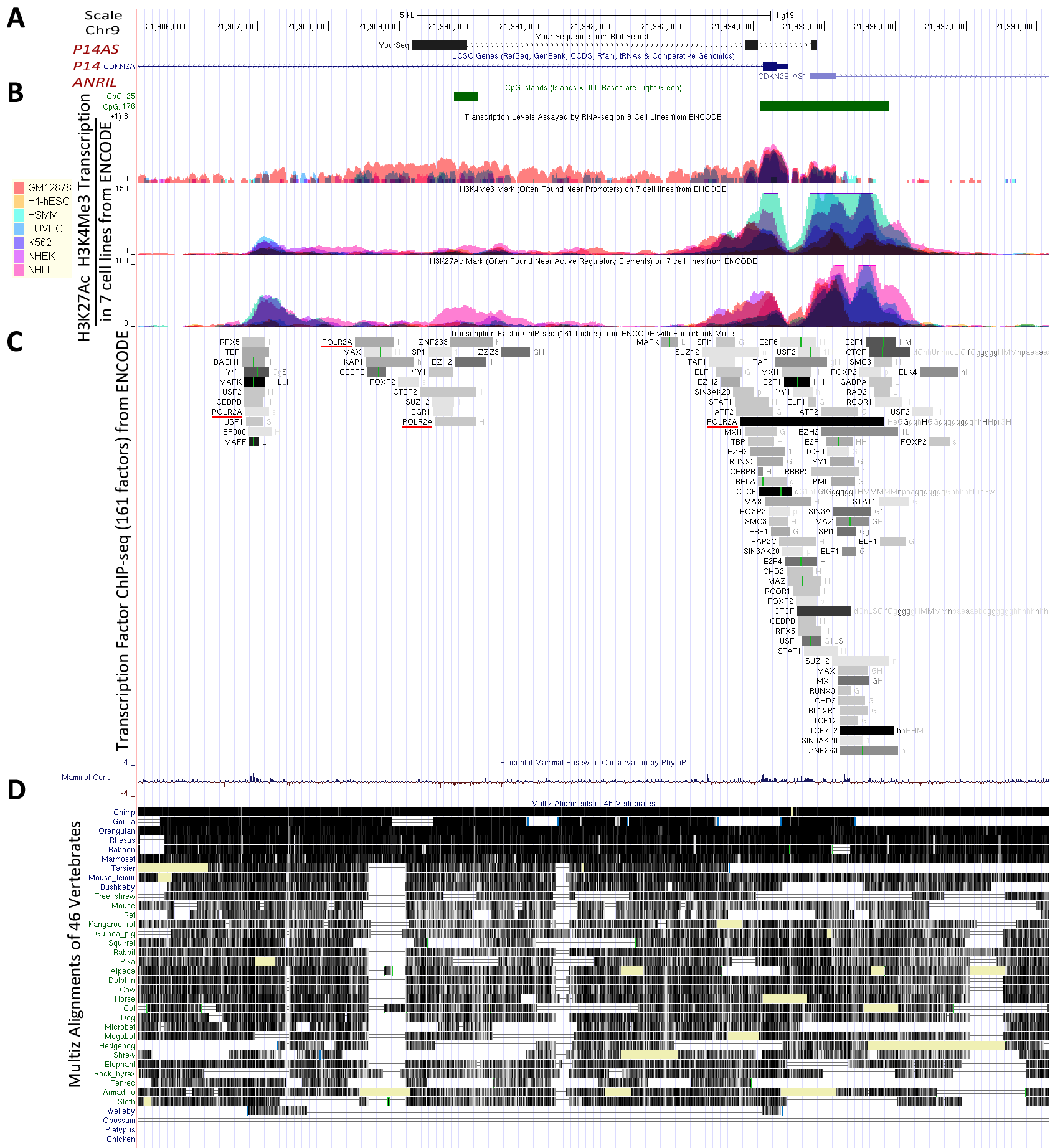


**Additional file 16: Fig. S8.** Graph of the *P14AS* gene in the *CDKN2A/B* locus. (**A**) CpG islands within the *CDKN2A/P14* gene. (**B**) The transcription and active histone modification status in the chromatin upstream of the *P14AS* gene in 7 cell lines from ENCODE. (**C**) Transcription factors binding to various fragments around the *P14AS* gene from ENCODE. The RNA polymerase II (POLR2A) is highlighted in red lines. (**D**) The conservation status of various fragments among vertebrates (adapted from the UCSC website).
