# Supplementary material for: Characterization of novel LncRNA P14AS as a protector of ANRIL through AUF1 binding in human cells
Source: Mol Cancer. 2020 Feb 27;19:42. doi: 10.1186/s12943-020-01150-4 (PMC7045492; doi:10.1186/s12943-020-01150-4)
Supplement: Supplementary file 17 — Additional file 17 Figure S9. Repression of P16, P14AS, and ANRIL expression in gastric cancer cells by engineered P16-specific DNA methyltransferase (P16-Dnmt). The gene expression level was detected using qRT-PCR assay. The detailed processes for the construction of P16-Dnmt and transfection experiments are previously described [27] [file 12943_2020_1150_MOESM17_ESM.docx]

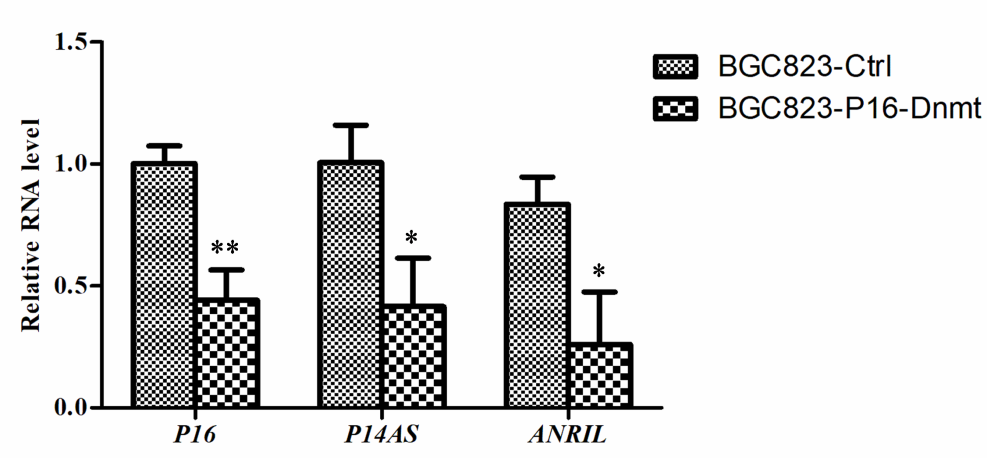


**Additional file 17: Fig. S9.** Repression of *P16*, *P14AS*, and *ANRIL* expression in gastric cancer cells by engineered *P16*-specific DNA methyltransferase (P16-Dnmt). The gene expression level was detected using qRT-PCR assay. The detailed processes for the construction of P16-Dnmt and transfection experiments are previously described [27]
